# A Transcription Factor Etv1/Er81 Is Involved in the Differentiation of Sweet, Umami, and Sodium Taste Cells

**DOI:** 10.1523/ENEURO.0236-22.2023

**Published:** 2023-04-21

**Authors:** Makoto Ohmoto, Masafumi Jyotaki, Karen K. Yee, Ichiro Matsumoto

**Affiliations:** Monell Chemical Senses Center, Philadelphia, PA 19104

**Keywords:** Etv1, salty, sweet, taste cell, umami

## Abstract

Taste cells are maintained by continuous turnover throughout a lifetime, yet the mechanisms of taste cell differentiation, and how taste sensations remain constant despite this continuous turnover, remain poorly understood. Here, we report that a transcription factor Etv1 (also known as Er81) is involved in the differentiation of taste cells responsible for the preference for sweet, umami, and salty tastes. Molecular analyses revealed that *Etv1* is expressed by a subset of taste cells that depend on Skn-1a (also known as Pou2f3) for their generation and express T1R genes (responsible for sweet and umami tastes) or *Scnn1a* (responsible for amiloride-sensitive salty taste). *Etv1^CreERT2/CreERT2^* mice express Etv1 isoform(s) but not Etv1 in putative proprioceptive neurons as comparable to wild-type mice, yet lack expression of Etv1 or an isoform in taste cells. These *Etv1^CreERT2/CreERT2^* mice have the same population of Skn-1a-dependent cells in taste buds as wild-type mice but have altered gene expression in taste cells, with regional differences. They have markedly decreased electrophysiological responses of chorda tympani nerves to sweet and umami tastes and to amiloride-sensitive salty taste evoked by sodium cation, but they have unchanged responses to bitter or sour tastes. Our data thus show that Etv1 is involved in the differentiation of the taste cells responsible for sweet, umami, and salty taste preferences.

## Significance Statement

The sense of taste plays important roles in food choice by detecting beneficial (i.e., nutritional) and potentially harmful substances. Senescent taste cells are replaced throughout an animal’s life by new ones generated from stem cells in the oral epithelia. However, the molecular mechanisms underlying taste cell turnover are poorly understood. By examining gene expression in taste cells and neural responses to taste substances, we found that the transcription factor Etv1 regulates the differentiation of sweet, umami, and salty taste cells. These findings advance our understanding of the molecular mechanisms underlying taste cell homeostasis and provide new insights into taste cell lineage.

## Introduction

The sense of taste plays a key role in food choice. Individual taste cells of vertebrate animals primarily respond to only one taste (Yarmolinsky et al., 2009; Matsumoto et al., 2013); thus, the diversity of tastes that an animal can perceive depends on the diversity of taste cells present. Mice can distinguish five basic tastes: sweet by cells co-expressing *Tas1r2* and *Tas1r3* or expressing *Tas1r3* alone, umami (i.e., savory) by cells that co-express *Tas1r1* and *Tas1r3*, bitter by *Tas2r*-expressing cells, sour by *Pkd2l1*-expressing cells, and salty by cells that express the amiloride-sensitive Na^+^ channel ENaC (Nelson et al., 2001, 2002; Zhao et al., 2003; Mueller et al., 2005; Huang et al., 2006; Chandrashekar et al., 2010; Ohmoto et al., 2020a). Taste cells can be distinguished from one another by their molecular features, especially by the expression of specific taste receptors, although no unique molecular markers specific for amiloride-sensitive salty taste cells (hereafter referred as sodium-taste cells) have been found, because ENaC subunit genes are also expressed in other taste cells (Chandrashekar et al., 2010; Nomura et al., 2020).

Taste cells emerge from local epithelial stem cells that express *Krt5* and *Sox2*, and their diverse populations are maintained by continuous turnover throughout an animal’s life (Beidler and Smallman, 1965; Farbman, 1980; Stone et al., 1995; Ohmoto et al., 2017, 2020b). The transcription factor Skn-1a (also known as Pou2f3) specifies the fate of a cell as a sweet, umami, bitter, or sodium taste cell lineage, rather than a sour lineage, probably at the late precursor stage (Matsumoto et al., 2011; Ohmoto et al., 2020a). However, the molecular and cellular mechanisms underlying how these cells then differentiate into different functional taste cells of the specified lineage remain elusive.

Etv1 (also known as Er81), a transcription factor of the ETS family, is one such candidate for this process. In the nervous system it is involved in the terminal differentiation of peripheral and central neurons, such as proprioceptive sensory neurons and cerebellar granule neurons (Arber et al., 2000; Patel et al., 2003; Abe et al., 2011; Fleming et al., 2016; Okazawa et al., 2016). Etv1 is also required for the survival of proprioceptive sensory neurons with various degrees of dependence among proprioceptive sensory neurons (Patel et al., 2003) but not for the survival of granule cells in cerebellum or their formation of synaptic connections (Abe et al., 2011).

*Etv1* is significantly enriched in taste buds of the rhesus macaque, and its expression is as high as that of taste-bud-specific transcription factor genes in tongue epithelium (Hevezi et al., 2009). Recent RNA sequencing data of taste cells support the presence of *Etv1* in *Tas1r3*^+^ sweet taste cells but not in bitter or sour taste cells (Lee et al., 2017; Sukumaran et al., 2017). However, it remains unclear whether sodium-taste cells or nonsensory taste bud cells express *Etv1* or whether all *Tas1r3*^+^ taste cells express it.

In the present study, we demonstrate that Etv1 is involved in differentiation of Skn-1a-dependent taste cells (i.e., often referred to as Type II cells): *Etv1* is expressed in sweet, umami, and sodium taste cells. Genetic ablation of Etv1 markedly decreases the expression of T1R genes in taste buds and culminates in the loss or reduction of responses to sweet and umami tastes in gustatory nerve recordings in mice. Etv1 ablation also represses *Scnn1a* expression and amiloride-sensitive salt taste responses in gustatory nerves. Sweet, umami, and sodium taste cells are still present in *Etv1*-deficient taste buds, but their populations are smaller than in heterozygous control mice.

## Materials and Methods

### Animals

C57BL/6J (stock #000664), homozygous B6.Cg-*Gt(ROSA)26Sor^tm14(CAG-tdTomato)Hze^*/J (*Rosa26^LSL-tdTomato^*, stock #007914; Madisen et al., 2010), and heterozygous B6(Cg)-*Etv1^tm1.1(cre/ERT2)Zjh^*/J (*Etv1^CreERT2/+^*, stock #013048; Taniguchi et al., 2011) strains were purchased from the Jackson Laboratory (for details of allele structure, see Fig. 2*C*). *Etv1^CreERT2/CreERT2^* (denoted as *Etv1^C/C^*) mice were generated by crossing heterozygous (i.e., *Etv1^CreERT2/+^*, denoted as *Etv1^C/+^*) males and females and maintained by crossing homozygous mice. *Skn-1a*^−/−^ mice with a C57BL/6J congenic background (i.e., B6.129-*Pou2f3^tm1Abek^*) were generated in a previous study (Ohmoto et al., 2020a) and maintained by crossing homozygous mice. *Etv1^C/C^* mice and *Rosa26^LSL-tdTomato^* (denoted as *R26^LSL-tdTom/LSL-tdTom^*) mice were mated to obtain *Etv1^C/+^*; *R26^LSL-tdTom/+^* mice, which were then mated with *Etv1^C/C^* mice to obtain *Etv1^C/C^*; *R26^LSL-tdTom/+^* mice. *Etv1^C/+^*; *R26^LSL-tdTom/+^* and *Etv1^C/C^*; *R26^LSL-tdTom/+^* mice that received tamoxifen, which caused tdTomato to label *Etv1*^+^ cells, are denoted as *Etv1^C/+^*; *R26^Tom/+^* and *Etv1^C/C^*; *R26^Tom/+^*, respectively. For embryo staging, the middle of the light period on the day of the vaginal plug was designated as embryonic day 0.5. All the mice used in this study had a C57BL/6J genetic background. Both sexes were used in all animal experiments, which were conducted according to a protocol approved by the Institutional Animal Care and Use Committee of the Monell Chemical Senses Center.

### Tamoxifen administration

Tamoxifen (10 mg/ml in corn oil; Sigma-Aldrich) was injected intraperitoneally in mice (100 mg/kg body weight) once a day for five consecutive days. The mice were euthanized 1 d after the last tamoxifen injection.

### Tissue preparation

Oral epithelia were dissected from mice deeply anesthetized by urethane, and sensory ganglia were dissected from mice that had been transcardially perfused with ice-cold PBS under anesthesia. Freshly dissected tissues were quickly embedded and frozen in optimal cutting temperature (O.C.T.) compound (Sakura Finetech). For tissues fixed with 4% paraformaldehyde (PFA), mice were deeply anesthetized with urethane and transcardially perfused with PBS followed by 4% PFA in PBS. For the developing neurons in the dorsal root ganglia (DRG), embryos were harvested from pregnant mice and fixed with 4% PFA, and lumber spinal columns including spinal cord and DRG were dissected. Dissected oral epithelia and embryonic spinal columns were postfixed, cryoprotected, and frozen as previously described (Ohmoto et al., 2008). Cryosections (8-μm thickness) were prepared using a CM1900 cryostat (Leica Microsystems), mounted on tissue adhesive-coated glass slides (Fisher Scientific), and preserved at −80°C until use.

### *In situ* hybridization

*In situ* hybridization using fresh-frozen or PFA-fixed sections was conducted as previously described (Ohmoto et al., 2008, 2020a). Digoxigenin-labeled and fluorescein-labeled antisense RNAs were synthesized and used as probes after fragmentation to ∼150 bases under alkaline conditions. The probe regions are shown in Table 1. For the *Tas2r* gene probes, riboprobes of *Tas2r104*, *Tas2r105*, *Tas2r118*, and *Tas2r126* were mixed. Fresh-frozen sections were fixed with 4% PFA and treated with diethylpyrocarbonate. The PFA-fixed sections were treated with proteinase K (3 μg/ml, Thermo Fisher Scientific), postfixed with 4% PFA, and then acetylated with acetic anhydride. The sections were prehybridized with salmon testis DNA, hybridized with the riboprobes for 40 h, and washed in 0.2× saline sodium citrate at 58°C, except when using the riboprobes for *Calhm1* and *Scnn1a*, for which sections were hybridized and washed at 65°C. After washing, chromogenic and/or fluorescent signals were developed as follows.

**Table 1:** Probes used for *in situ* hybridization analyses

Gene name	Accession no.	Probe region
*Etv1*	BC005645	1–2486
*Tas1r1*	AF337040	765–2761
*Tas1r2*	AF337041	641–2804
*Tas1r3*	AF337039	525–2725
*Tas2r104*	BC148229	1–921
*Tas2r105*	AF227147	1–903
*Tas2r118*	BC104401	1–900
*Tas2r126*	NM_207028	73–899
*Scnn1a*	BC133688	913–2333
*Gnat3*	AK040065	41–1019
*Plcb2*	BC145249	588–3123
*Trpm5*	AF228681	310–3491
*Calhm1*	LC270870	1–1407, 2148–2369
*Calhm3*	LC270871	1–1653
*Pkd2l1*	NM_181422	226–3275
*Entpd2*	NM_009849	20–1822

For single-label *in situ* hybridization, the chromogenic signals of the hybridized probes were detected using alkaline phosphatase-conjugated anti-digoxigenin antibodies (Roche Diagnostics, 11093274910, RRID:AB_514497) followed by 4-nitro blue tetrazolium chloride/5-bromo-4-chloro-3-indolyl phosphate as a substrate. The signals were developed overnight except for *Calhm1* probes, which were developed for 2 d. Short-term signal development for *Tas1r3* was also investigated at 4 h. Stained images were obtained using a Nikon Eclipse 80i microscope equipped with a DXM1200C digital camera (Nikon Instruments).

For double-fluorescence *in situ* hybridization in oral epithelia, the fluorescent signals of the riboprobes were developed using alkaline phosphatase-conjugated anti-digoxigenin antibodies followed by the HNPP Fluorescent Detection Set (Roche Diagnostics) and biotin-conjugated anti-fluorescein antibodies (Vector Laboratories, BA-0601, RRID:AB_2336069) followed by avidin-biotin complex (Vector Laboratories), TSA Biotin Tyramide Reagent (PerkinElmer), and Alexa488-conjugated streptavidin (Thermo Fisher Scientific). Fluorescent images were acquired with a TCS SP2 confocal microscope (Leica Microsystems). Brightness and contrast were linearly adjusted, and all adjustments were globally applied to confocal images and overlaid multiple fluorescence images using Photoshop (Adobe Systems). The presence of fluorescence signals was determined manually by visual inspection.

For double-fluorescence *in situ* hybridization in geniculate ganglia (GG), fluorescein-labeled probes and digoxigenin-labeled probes were sequentially detected as follows. Sections were incubated with biotin-conjugated anti-fluorescein antibodies, followed by avidin-biotin complex, TSA-plus Biotin solution (PerkinElmer), and Alexa488-conjugated streptavidin. After treatment with 3% H_2_O_2_ in PBS for 60 min, sections were then incubated with horseradish peroxidase-conjugated anti-digoxigenin antibodies (Roche, 11207733910, RRID:AB_514500) followed by TSA-plus Cyanine 3 solution (PerkinElmer) and 4',6-diamidino-2-phenylindole (DAPI). The fluorescent images were acquired, and optical confocal images were processed as described above. Only neurons whose profiles contain a nucleus with and without fluorescence signals were counted, and the frequencies of expression of *Etv1*-derived transcripts in *P2x2*^+^ neurons and those of *P2x2*^+^ neurons in GG were quantitatively analyzed using every 10 sections (i.e., 80 μm apart) of the GG of wild-type and *Etv1^C/C^* mice (*n *=* *3 for each genotype).

Double labeling of *Scnn1a* with other genes with a combination of chromogenic and fluorescent signals was conducted as previously described (Taruno et al., 2013; Ohmoto et al., 2020a). The fluorescent signals were first developed using a biotin-conjugated anti-fluorescein antibody (Vector Laboratories) followed by avidin-biotin complex (Vector Laboratories), TSA Biotin Tyramide Reagent (PerkinElmer), and Alexa488-conjugated streptavidin (Thermo Fisher Scientific). After acquiring the fluorescent images, the chromogenic signals for *Scnn1a* were detected using an alkaline phosphatase-conjugated anti-digoxigenin antibody and 4-nitroblue tetrazolium chloride/5-bromo-4-chloro-3-indolyl-phosphate. The signals were developed for 7 d. Stained images were obtained as described above. Fluorescent and stained images were processed with Photoshop (Adobe Systems) and analyzed on a computer screen. Fluorescence signal and chromogenic stain surrounding a nucleus were counted as positive signal. Sporadic punctate fluorescence was regarded as nonspecific background. The frequencies of expression of *Scnn1a* in *Skn-1a*^+^ cells (i.e., sodium taste cells) were semiquantitatively analyzed by calculating the ratios of taste bud profiles that harbored at least one cell co-expressing *Skn-1a* and *Scnn1a* to all taste bud profiles using every 8 and 10 sections of palate and fungiform papillae (FuP), respectively, of wild-type and *Etv1^C/C^* mice (*n *=* *3 for each genotype). *Scnn1a*^+^ cells devoid of *Skn-1a* signal, which are likely sour taste cells (Chandrashekar et al., 2010; Ohmoto et al., 2020a), were not counted.

### Quantitative PCR

Epithelia of CvP, anterior tongue, and soft palate, and DRG and GG were dissected from C57BL/6J mice and *Etv1^C/C^* mice. Total RNA was extracted using TRIzol (Life Technologies, 15596026), and cDNA was synthesized using SuperScript IV VILO Master Mix with ezDNase Enzyme (Life Technologies, 11766050). Quantitative PCR (qPCR) was performed using Fast SYBR Green Master Mix kit (Applied Biosystems, 4385612). Genes that did not show single peaks of melt curve in a C57BL/6J tissue were regarded as “not amplified” and removed from further analyses. *Gapdh* was used as a control to normalize the expression levels of individual genes. The relative gene expression level was calculated as 2^-(CTTarget-CTGapdh)^ (Livak and Schmittgen, 2001). Details about the primers used are given in Table 2.

**Table 2:** Primers used for qPCR analyses

Gene	Accession no.	Amplified region	Forward primer (5′–>3′)	Reverse primer (5′–>3′)
*Gapdh*	BC085315	735–843	GCATGGCCTTCCGTGTTCCTA	GATGCCTGCTTCACCACCTTCT
*Etv1*	L10426	221–392	GCAAGTGCCTTACGTGGTCAC	AGGTACCTGAGCTTCAGCAAG
		1320–1517	CGTTGGGGCATTCAGAAGAAC	TTCCATGTCCGTCTTCAGCAG
*Tas1r1*	AF337040	1651–1795	ACAATCAGGTGCCTGTGTCAG	TGGCAGGTGTGAAGCTCACTC
*Tas1r2*	AF337041	1284–1446	TCGTCTATCCATGGCAGCTAC	TAGGAGGCGATGCTTTGGAAG
*Tas1r3*	AF337039	481–652	ACCTTCAACGGCACCCTTCAG	CAGGTGAAGTCATCTGGATGC
*Gnat3*	BC147839	530–679	GACTTAGACAGACTCACAGCC	CTCTGATCTCTGGCCACCTAC
*Plcb2*	NM_177568	1731–1857	CCTGGAGGTGACAGCTTATGA	GAGCTCCGTGAAGGAAGAGAC
*Trpm5*	AF228681	3320–3491	GGAAAACGGCACACAGAGTGG	CCACAGTTCTGAGAGCTTGAG
*Calhm1*	LC270870	535–665	TTGCGCTGCATCTCTCAGGCA	TAGTGGGACCAGTACTTGCTC
*Calhm3*	LC270871	453–571	GCAAGTGCAGCTCTTCCTAGC	TCCAGCCGATGGCCTGTGACA
*Tas2r104*	BC148229	488–635	ACATCCTGGCTGATCCACCTG	ATTGCATCTGCCTGCTGTGTC
*Tas2r105*	BC125541	160–318	TGACTGGCTTCCTTCTCATCG	TCAGGTGATTCACAGTCATCC
*Tas2r118*	BC104401	298–411	GCTGTCCTCTACTGTGTCAAG	AGAAGCTATCAGAGCACCCAG
*Tas2r126*	NM_207028	511–678	AAGGAGTGGAACAGAAGGCTG	GTTGGGGTCTTGCAAGCTGTG
*Scnn1a*	NM_011324	2107–2259	GTCTGTGAAGTCCCAGGATTG	ACCATCGTGACAGAGGGAGAC
*Pkd2l1*	NM_181422	1715–1921	TGCCATCGACAATGCCAACAG	CGCAGCCTTAGTAGGGTCTTG
*Entpd2*	NM_009849	395–560	CATGCGCCTACTCAACCTGAC	GTTCTCCAGCAGGTAGTTGGC

### 5’-RACE

First strand cDNA to *Etv1* gene-derived transcript in the total RNA extracted from DRG of C57BL/6J mice was synthesized after the treatment with ezDNase Enzyme (Life Technologies, 11766050) using a primer 5′-TTCCATGTCCGTCTTCAGCAG-3′, which is also used to amplify C-terminal coding region of Etv1 by qPCR, and then poly-adenylated by terminal deoxynucleotidyl transferase. A 5′ end of *Etv1* gene-derived transcript was amplified by PCR using a primer 7R1 (5′-CGGTCAGGTTTCGGAGTATGAGC-3′) and oligo(dT) primer and then a primer 7R2 (5′- ATAGTCGACCACTTGTGGCTTCTGATCATAGGC-3′) and oligo(dT) primer. Sequence of the amplified cDNA fragment was determined using the primer 7R2.

### Immunohistochemistry

Immunohistochemical analyses using 4% PFA-fixed sections were conducted as previously described (Ohmoto et al., 2008). The sections were treated in a preheated target retrieval solution (pH 9; Agilent Technologies) at 80°C for 20 min before blocking. Primary antibodies used were rabbit anti-Etv1 (1:500, Abcam, ab81086, RRID:AB_1640495), rabbit anti-Trpm5 (1:3000, Alomone Labs, ACC-045, RRID:AB_2040252), mouse anti-IP_3_R3 (1:1000, BD Biosciences, 610312, RRID:AB_397704), goat anti-KCNQ1 (1:300, Santa Cruz Biotechnology, sc-10 646, RRID:AB_2131554), rabbit anti-Ddc (1:2000, GeneTex, GTX30448, RRID:AB_367199), goat anti-parvalbumin (1:500, Swant, PVG-213, RRID:AB_2650496), and rabbit anti-P2X2 (1:500, Sigma-Aldrich, P7982, RRID:AB_261187) antibodies. For single staining, signals were developed using a biotin-conjugated anti-rabbit IgG antibody (1:500, Vector Laboratories, BA-1000, RRID:AB_2313606) followed by avidin-biotin complex (Vector Laboratories) and the Metal Enhanced DAB Substrate kit (Thermo Fisher Scientific). For fluorescent labeling, Alexa 488-conjugated, 555-conjugated, and 647-conjugated antibodies [1:500, Thermo Fisher Scientific, A11029 (RRID:AB_138404), A11034 (RRID:AB_2576217), A11055 (RRID:AB_2534102), A21206 (RRID:AB_2535792), A21245 (RRID:AB_141775), A21432 (RRID:AB_2535853), A21447 (RRID:AB_141844)] were used as the secondary antibodies. Stained and fluorescent images were acquired and processed as described above.

Fluorescent labeling of KCNQ1 and double-fluorescence labeling of IP_3_R3 and Ddc (also known as AADC) were conducted using every 12, 6, and 6, sections of CvP, FuP, and palate, respectively, of *Etv1^C/+^*; *R26^Tom/+^* (heterozygous control) and *Etv1^C/C+^*; *R26^Tom/+^* mice (*n = *3 for each genotype). The sections were counterstained with DAPI, and fluorescent images were acquired with a confocal microscope. KCNQ1^+^ cells, Ddc^+^ cells, IP_3_R3^+^ cells, and IP_3_R3^+^tdTomato^+^ cells that had DAPI signals in taste bud profiles were counted, and the ratios of IP_3_R3^+^tdTomato^+^ cells to IP_3_R3^+^ cells were calculated.

Double-fluorescence labeling of P2X2 and KCNQ1 was conducted in gustatory tissues of wild-type and *Etv1^C/C^* mice (*n = *2 for each genotype). The sections were counterstained with DAPI, and fluorescent images were acquired with a confocal microscope in a Stellaris 5 WLL AOBS system (Leica Microsystems). Serial z-stacks were merged as maximum projection images using LASX software.

### Whole chorda tympani nerve recordings

We investigated the electrophysiological response of the chorda tympani nerve in mice of the wild-type and *Etv1^C/C^* mice at two to four months old of both sexes (*n *=* *8 per genotype). The experimenters were blinded to the genotype of the mice during testing. The mice were anesthetized with an intraperitoneal injection of a mixture of 4.28 mg/ml ketamine, 0.86 mg/ml xylazine, and 0.14 mg/ml acepromazine in saline (5 μl/g body weight). Anesthesia was maintained with additional injections. Each mouse was fixed with a head holder after its trachea was cannulated; then the chorda tympani nerve was dissected free from the lingual nerve near the tympanic bulla, cut, and placed on a platinum wire recording electrode. An indifferent electrode touched the walls of the wound. Taste stimuli were delivered to the tongue with a computer-controlled open flow system under constant flow and temperature (25°C) conditions. Each stimulation lasted for 30 s with a 60-s rinse between stimulations. Care was taken to ensure the flow was directed over the FuP. The nerve impulses were fed into a custom-made amplifier, monitored over a loudspeaker and with an oscilloscope, and recorded (PowerLab/sp4; AD Instruments). The integrated response during stimulation was calculated by subtracting the area of nerve activity preceding the stimulation from that during stimulation. Thus, the data reflect the level of activity during the stimulation period. The responses to all compounds were expressed relative to the response to 0.1 m NH_4_Cl for each mouse. The averages for each animal and group were calculated for the statistical analyses.

### Statistical analyses

Data are shown as the mean ± SD except those of gustatory nerve recordings that are shown as the mean ± SEM. A Welch’s *t* test (for qPCR, taste bud numbers, taste bud cell populations, and tdTomato^+^ cell ratio to IP_3_R3^+^ taste cell) or repeated-measures two-way ANOVA (for gustatory nerve recordings) was used to determine the effects of genotype using Prism 6 and 8 software (GraphPad Software). Sidak’s multiple comparison tests were conducted to identify significant differences between pairs of mean values.

## Results

### Expression of *Etv1* in taste cells

**Figure 1.**
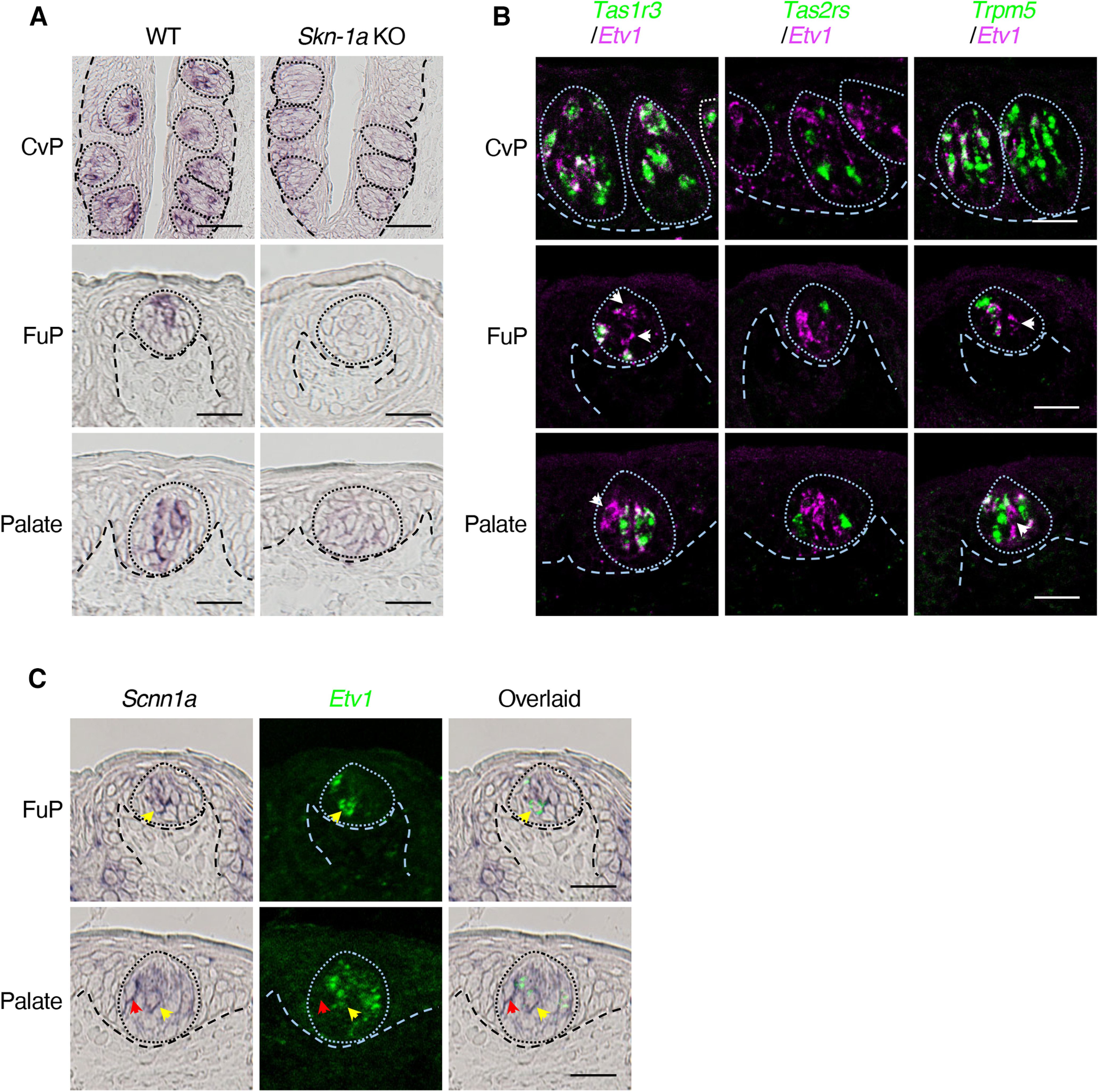
*Etv1* is expressed in a subset of Skn-1a-dependent taste cells. ***A***, Single-color *in situ* hybridization studies to investigate the expression of *Etv1* mRNA in taste buds in circumvallate (CvP; top) and fungiform (FuP; middle) papillae and in palate (bottom) of wild-type (WT; left) and *Skn-1a*^−/−^ (right) mice. *n = *3 for CvP, *n = *1 for palate and FuP; *n *≥ 9 sections for each experiment. Scale bars: CvP, 50 μm; FuP and palate, 25 μm. ***B***, Double-fluorescence *in situ* hybridization of *Etv1* (magenta) with marker genes (green) in the CvP (top), FuP (middle), and palate (bottom) taste buds in WT mice. White arrowheads indicate cells singly expressing *Etv1*. For each experiment: CvP, *n *≥* *2, with ≥7 sections; FuP, *n *≥* *2, with ≥18 sections; palate, *n *≥* *1, with ≥9 sections. Scale bars: 25 μm. ***C***, Double-labeling *in situ* hybridization to study expression of *Scnn1a* in *Etv1*^+^ taste cells: *Scnn1a* (dark purple; left), *Etv1* (green; middle), and merged (right) images of FuP (top) and palate (bottom) taste buds of WT mice. Yellow arrowheads indicate cells co-expressing *Scnn1a* and *Etv1*; red arrowheads indicate cells expressing only *Scnn1a*. *n = *3 for each experiment. Scale bars: 25 μm. Taste buds and basal epithelial-mesenchymal boundaries are indicated by broken lines.

We examined the expression of *Etv1* mRNA in mouse taste buds by *in situ* hybridization (for probe lengths and regions, see Table 1) and observed signals in a minor subset of taste bud cells in all gustatory areas of wild-type mice but not in *Skn-1a*-deficient mice, which indicates the restricted expression of *Etv1* mRNA in Skn-1a-dependent taste cells (Fig. 1*A*). Double-fluorescence *in situ* hybridization analyses showed that *Etv1* is expressed in a subset of *Skn-1a*^+^ and *Trpm5*^+^ (i.e., sweet, umami, and bitter taste) cells in the CvP in the wild-type mice, especially in *Tas1r3*^+^ cells, but not in *Tas2r*^+^ cells responsible for bitter taste (Fig. 1*B*). In taste buds in the FuP and palate, *Etv1* was present in the cells lacking *Tas1r3*, *Tas2r*, and *Trpm5* expression in addition to *Tas1r3*^+^ cells, suggesting its expression in sodium-taste cells. Double-label *in situ* hybridization of *Etv1* with *Scnn1a* showed partial co-expression in taste cells in the FuP and palate (Fig. 1*C*). Together with its restricted expression to Skn-1a-dependent taste cells, these results indicate that *Etv1* is expressed in *Skn-1a*^+^*Scnn1a*^+^ sodium-taste cells (Ohmoto et al., 2020a). Therefore, gene expression analyses demonstrate that *Etv1* is expressed in sweet, umami, and sodium taste cells, all of which are involved in eliciting taste preferences.

### *Etv1* deficiency in taste buds of *Etv1^C/C^* mice

**Figure 2.**
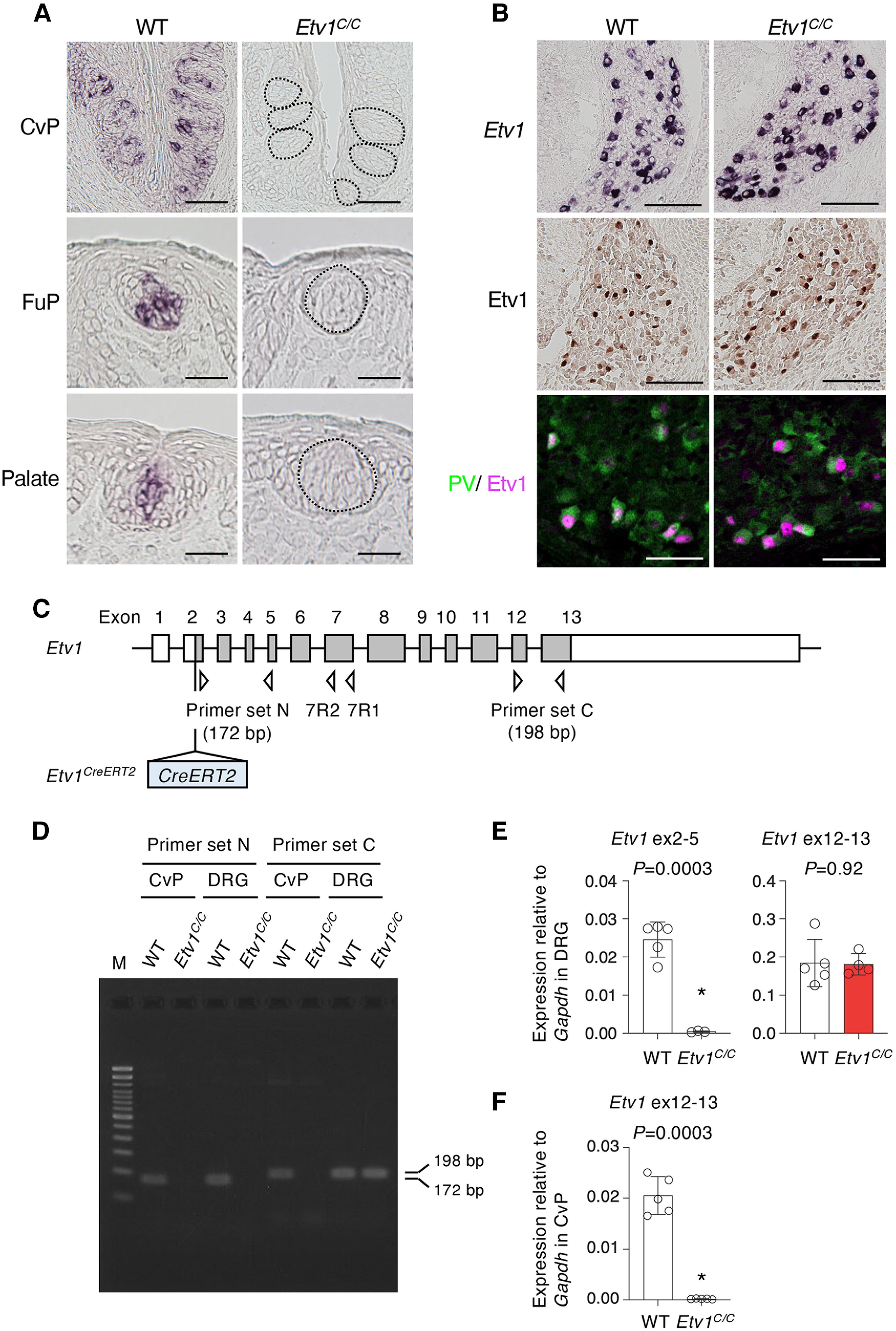
Loss of expression of *Etv1* mRNA in taste buds of *Etv1^C/C^* mice. ***A***, Single-color *in situ* hybridization studies to investigate the expression of *Etv1* mRNA in taste buds in circumvallate (CvP; top) and fungiform (FuP; middle) papillae and in palate (bottom) of wild-type (WT; left) control and *Etv1^C/C^* (right) mice. Taste buds in *Etv1^C/C^* mice are indicated by broken lines. *n = *3 for CvP, *n = *1 for FuP and palate; ≥9 sections for each experiment. Scale bars: CvP, 50 μm; FuP and palate, 25 μm. ***B***, Expression of mRNA (top) and protein (middle and bottom) of Etv1 isoform(s) in the sensory neurons in the dorsal root ganglia of wild-type (WT; left) and *Etv1^C/C^* (right) embryos at embryonic day 15.5. Etv1-immunoreactive signals (magenta) were observed in the parvalbumin (PV, green)-expressing proprioceptive neurons (bottom). *n = *3 for each experiment, 10 sections for each genotype. Scale bars: 100 μm (chromogenic images) and 50 μm (fluorescent images). ***C***, Schematic structure of *Etv1* gene and its *CreERT2* knock-in allele. Boxes indicate exons. Protein coding regions are shaded. Primers used for PCR analyses are indicated by triangles (see Table 2). The reverse primer of the primer set C and primers 7R1 and 7R2 were used for 5’-RACE. *CreERT2* sequence is inserted in front of the forward primer of the primer set N in the second exon of *Etv1* (Extended Data Fig. 2-1). ***D***, Gel image of agarose electrophoresis of qualitative PCR products from circumvallate papillae (CvP) and dorsal root ganglia (DRG) of wild-type (WT) and *Etv1^C/C^* mice. Predicted amplicon sizes by primer sets N and C are 172 and 198 bp, spanning from exon 2 to exon 5 and from exon 12 to exon 13, respectively. Lane M, 100-bp DNA ladder marker. ***E***, ***F***, qPCR analyses to detect the expression of *Etv1* and/or *Etv1*-derived transcripts in DRG (***E***) and CvP (***F***) in WT and *Etv1^C/C^* mice. Open circles, individual data; white and red bars are the mean ± SD of WT and *Etv1^C/C^* mouse tissues, respectively. Significance was assessed by Welch’s *t* test. Primers used are sets N and C to DRG (***E***) and set C to CvP (***F***). *n *=* *5 for WT mice; *n *=* *4 for DRG and *n *=* *5 for CvP for *Etv1^C/C^* mice.

Mutant mouse strains with a null or loss-of-function mutation for Etv1 are useful models to study the function of Etv1 in the taste system. *Etv1^C/C^* mice are designed to encode a tamoxifen-inducible Cre recombinase (CreER) instead of Etv1 and carry a *CreERT2-frt* sequence in exon 2 in place of the initial ATG of the *Etv1* coding sequence (Extended Data Fig. 2-1*A*; Taniguchi et al., 2011). Therefore, they were expected to be conventional knock-outs like *Etv1^nlslacZ^* mice (Extended Data Fig. 2-1*A*; Arber et al., 2000). *Etv1^C/C^* mice, however, are viable and fertile and do not present any overt abnormalities, although other Etv1-null mutant strains (*Etv1^ETS/ETS^* and *Etv1^nlslacZ/nlslacZ^* mice; for their allele structures, see Extended Data Fig. 2-1*A*) exhibit limb ataxia or abnormal postures attributable to defects in proprioceptive function and die three to five weeks after birth (Arber et al., 2000; Patel et al., 2003). Therefore, we first evaluated whether *Etv1* is knocked out in *Etv1^C/C^* mice. *Etv1* mRNA was completely absent in taste buds in all gustatory areas examined in *Etv1^C/C^* mice, while taste buds of wild-type mice showed *Etv1* mRNA signals (Fig. 2*A*). Intriguingly, however, we observed the signals of *Etv1* mRNA and protein in a subset of dorsal root ganglia (DRG) neurons of wild-type and *Etv1^C/C^* mice (Fig. 2*B*); the frequency and intensity of these signals in *Etv1^C/C^* mice were qualitatively comparable to those in wild-type mice. Etv1 immunoreactivity was observed in most parvalbumin^+^ neurons (i.e., proprioceptive sensory neurons) in both wild-type and *Etv1^C/C^* mice (Fig. 2*B*). These results indicate that the *Etv1^C/C^* mice are Etv1 null mutant in taste buds and suggest that Etv1 variant(s) are expressed in DRG neurons.

10.1523/ENEURO.0236-22.2023.f2-1 Extended Data Figure 2-1 Schematic structures of *Etv1* alleles and their transcripts. ***A***, Schematic gene structures of wild-type and gene-targeted *Etv1* alleles. Initial methionine codon of *Etv1* gene is replaced by *CreERT2* and *nlslacZ* together with *PGK-Neo* in *Etv1^CreERT2^* and *Etv1^nlslacZ^* alleles, respectively (Arber et al., 2000; Taniguchi et al., 2011). *Etv1^ETS^* allele retains *IRES-taulacZ and PGK-Neo* in exon 11 to disrupt the ETS domain of Etv1 (Arber et al., 2000). ***B***, Etv1 gene-derived transcripts in DRG. *Etv1* mRNA was detected by qualitative and quantitative PCR, but 5’-RACE primarily amplified *Etv1*-v2 mRNAs. ***C***, *Etv1^CreERT2^* allele-derived transcripts in DRG. Etv1-like immunoreactivity and amplification of C-terminal coding region by PCR in *Etv1^C/C^* mice can be explained by the presence of Etv1 isoform b encoded by *Etv1*-v2 mRNA. Download Figure 2-1, TIF file.

To determine whether Etv1 is knocked out and whether an Etv1 variant is expressed in DRG of *Etv1^C/C^* mice, we performed qualitative PCR and qPCR using primers corresponding to N-terminal and C-terminal coding sequences. cDNA fragments corresponding to N-terminal coding sequence were not amplified from CvP or DRG of *Etv1^C/C^* mice, indicating the disruption of *Etv1* by the insertion of *CreERT2* in DRG as well as CvP (Fig. 2*C–F*; Extended Data Fig. 2-1). However, cDNA fragments corresponding to the ETS domain in the C-terminal region were amplified from DRG but not from CvP of *Etv1^C/C^* mice (Fig. 2*C–F*; Extended Data Fig. 2-1*A*). 5’-RACE revealed that a major transcript in DRG is *Etv1* variant 2 (*Etv1*-v2, NM_001163154), which has little impact of its expression by CreERT2 insertion (Fig. 2*C*; Extended Data Fig. 2-1*B*,*C*). These results indicate that the *Etv1^C/C^* mice are Etv1 null mutant in taste buds and that the major transcript from the *Etv1* allele in taste tissues is *Etv1*. In DRG, *Etv1*-v2 and its encoded protein, Etv1 isoform b, are expressed in DRG neurons, in addition to Etv1 mRNA and protein, in proprioceptive neurons. It remains unclear whether the Etv1 isoform b is as functional as Etv1, although it can seemingly compensate for the loss of Etv1 in the proprioceptive neurons.

### Involvement of Etv1 in the expression of taste receptor genes in *Etv1*^+^ cells in taste buds

**Figure 3.**
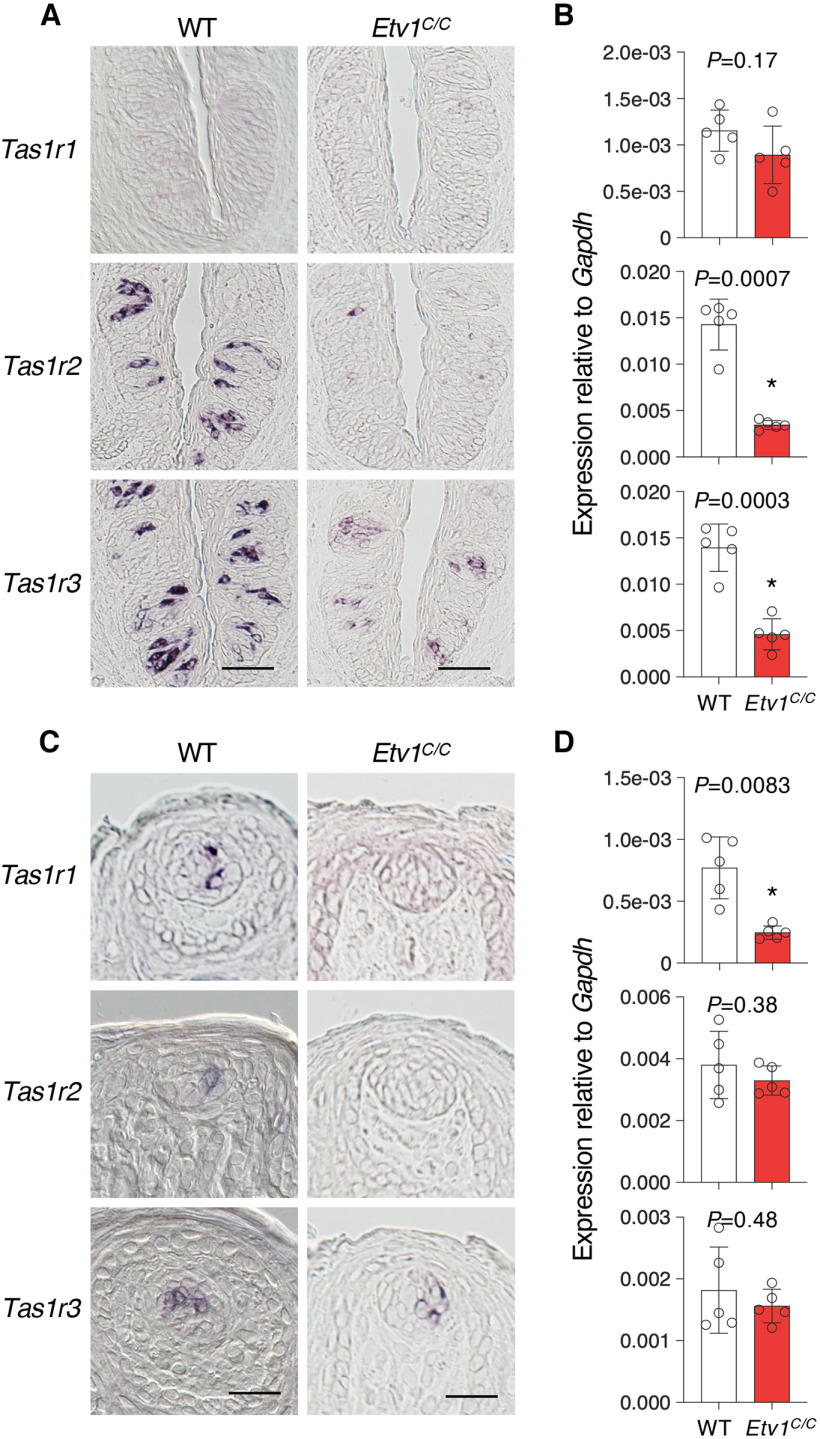
Etv1 regulates the expression of T1R genes in taste cells. The expression of T1R taste receptor genes in taste buds was analyzed in circumvallate (***A***, ***B***) and fungiform (***C***, ***D***) papillae and soft palate (Extended Data Fig. 3-1) of wild-type (WT) and *Etv1^C/C^* mice. ***A***, ***C***, *In situ* hybridization analyses. Left, WT mice (*n = *3; ≥9 sections for CvP, ≥27 sections for FuP). Right, *Etv1^C/C^* mice (*n = *3; ≥9 sections for CvP, ≥27 sections for FuP). Scale bars: CvP, 50 μm and FuP, 25 μm. ***B***, ***D***, qPCR analyses. *n *=* *5 for each genotype. Open circles, individual data; white and red bars are the mean ± SD of WT and *Etv1^C/C^* tissues, respectively. Significance was assessed by Welch’s *t* test.

To understand the role of Etv1 in taste cells, we studied the impact of *Etv1* deficiency on taste receptor gene expression by *in situ* hybridization and qPCR analyses in taste buds of *Etv1^C/C^* mice. The umami taste receptor gene *Tas1r1*, which is predominantly expressed in the taste buds in FuP and palate but rarely detected in CvP of wild-type mice, as reported previously (Hoon et al., 1999; Nelson et al., 2001), was completely absent from the taste buds of any gustatory area of *Etv1^C/C^* mice (Fig. 3*A*,*C*; Extended Data Fig. 3-1*A*). The sweet taste receptor gene *Tas1r2*, which is predominantly expressed in the taste buds in CvP and palate but rarely detected in FuP of wild-type mice, as reported previously (Hoon et al., 1999; Nelson et al., 2001), was almost (in CvP) or completely (in FuP and palate) lost in taste buds of *Etv1^C/C^* mice (Fig. 3*A*,*C*; Extended Data Fig. 3-1*A*). qPCR analyses exhibited results consistent with *in situ* hybridization analyses but found no significant differences where *Tas1r1* and *Tas1r2* are rarely expressed in wild-type mice (Fig. 3*B*,*D*; Extended Data Fig. 3-1*B*). The sweet and umami taste receptor gene *Tas1r3* was less influenced by *Etv1* deficiency than were *Tas1r1* and *Tas1r2*: intensity and frequency of signals of *Tas1r3* in taste buds were weaker and sparser, respectively, than in wild-type mice (Fig. 3*A*,*C*; Extended Data Fig. 3-1*A*). qPCR showed drastic decrease in *Tas1r3* expression only in CvP of *Etv1^C/C^* mice (Fig. 3*B*,*D*; Extended Data Fig. 3-1*B*). However, *Tas1r3* signals were barely detectable in the taste buds in FuP and palate of *Etv1^C/C^* mice when signal was developed for a shorter time (Extended Data Fig. 3-1*C*,*D*), suggesting a decrease in *Tas1r3* expression by *Etv1* deficiency.

10.1523/ENEURO.0236-22.2023.f3-1 Extended Data Figure 3-1 Etv1 regulates the expression of T1R genes in taste cells in palate**. *A***, *In situ* hybridization demonstrates impact of *Etv1* deficiency on expression of T1R taste receptor genes in taste buds in soft palate. Signals were developed overnight. Left, Wild-type (WT) mice (*n = *3). Right, *Etv1^C/C^* mice (*n = *3). Scale bars: 25 μm. ***B***, qPCR analyses of T1R genes in wild-type control (WT) and *Etv1^C/C^* mice. *n *=* *5 for each genotype. Open circles, Individual data. White and red bars are the mean ± SEM of WT and *Etv1^C/C^* tissues, respectively. Significance was assessed by Welch’s *t* test. ***C***, ***D***, *In situ* hybridization demonstrates impact of *Etv1* deficiency on expression of *Tas1r3* in taste buds in soft palate (***C***) and fungiform papillae (***D***). Signals were developed for 4 h. Left, WT mice (*n = *3). Right, *Etv1^C/C^* mice (*n = *3). Scale bars: 25 μm. Download Figure 3-1, TIF file.

**Figure 4.**
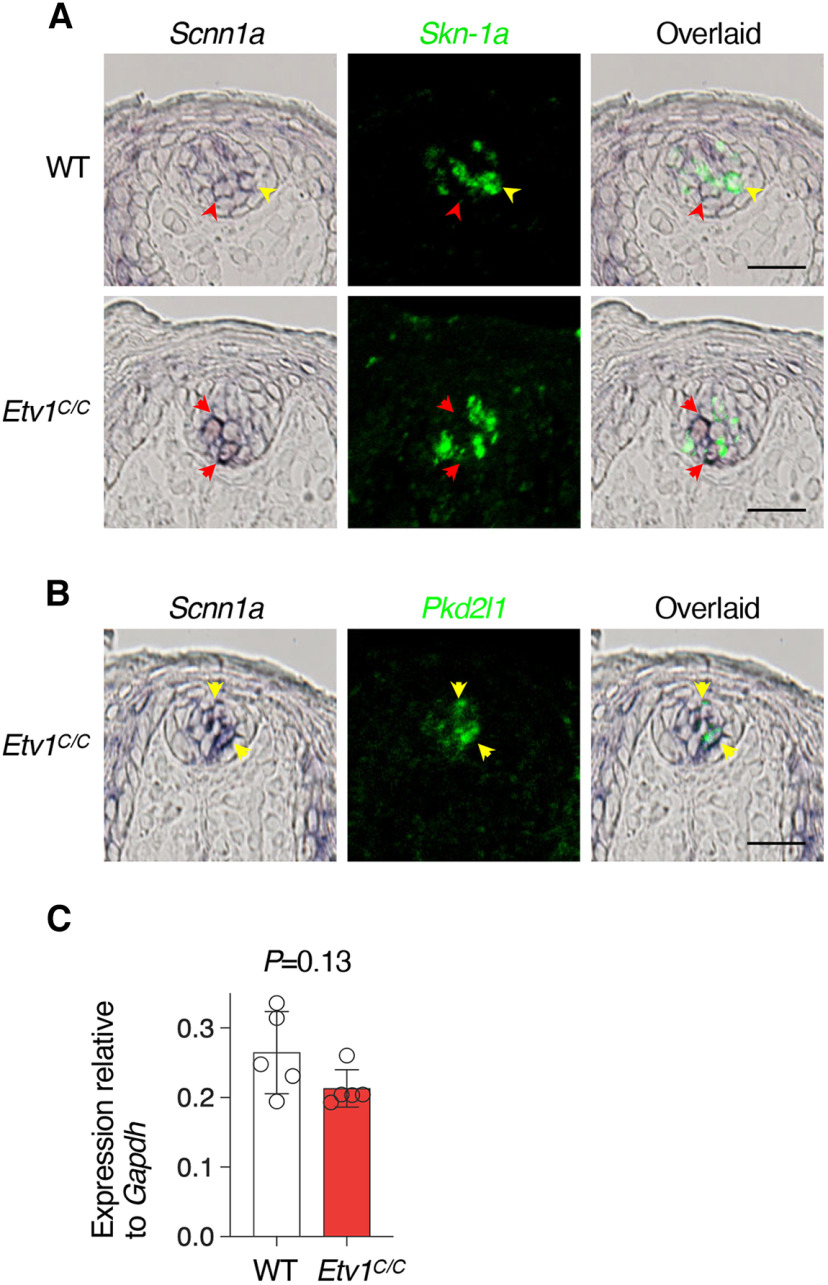
Etv1 positively regulates expression of the *Scnn1a* sodium taste receptor gene. ***A***, ***B***, Double-label *in situ* hybridization demonstrates expression of *Scnn1a* in *Skn-1a*^+^ taste cells (***A***) and in *Pkd2l1*^+^ taste cells (***B***). Shown are *Scnn1a* (dark purple; left), *Skn-1a* or *Pkd2l1* (green; middle), and overlaid (right) images of taste buds in fungiform papilla of wild-type (WT) and *Etv1^C/C^* mice. Yellow arrowheads indicate cells co-expressing *Scnn1a* with *Skn-1a* (***A***) or *Pkd2l1* (***B***), and red arrowheads indicate cells expressing only *Scnn1a*. *n = *3; 27 sections for each genotype (***A***); *n = *2; 18 sections (***B***). Scale bars: 25 μm. ***C***, qPCR analysis of *Scnn1a* in taste buds in fungiform papillae of WT and *Etv1^C/C^* mice. *n *=* *5 for each genotype. Open circles, individual data; white and red bars are the mean ± SD of WT and *Etv1^C/C^* tissues, respectively. Significance was assessed by Welch’s *t* test.

*Scnn1a*, a gene responsible for amiloride-sensitive salty taste, is expressed in all *Pkd2l1*^+^ cells and a subset of *Skn-1a*^+^ cells in taste buds in the FuP in wild-type mice, as reported previously (Chandrashekar et al., 2010; Ohmoto et al., 2020a). In *Etv1^C/C^* mice, *Scnn1a* was rarely co-expressed with *Skn-1a* in *Etv1^C/C^* mice (Fig. 4*A*), while its co-expression with *Pkd2l1*^+^ in sour taste cells was unaltered (Fig. 4*B*), indicating that Etv1 is required for the *Scnn1a* expression in sodium taste cells. However, qPCR analyses exhibited higher relative expression of *Scnn1a* than of T1R genes in FuP and did not show significant differences between wild-type and *Etv1^C/C^* mice (Fig. 4*C*). *Scnn1a* may be expressed in many cells inside and/or outside taste buds at lower levels than in sour and sodium taste cells, and thus the decrease in sodium taste cells by *Etv1* deficiency may be too small to affect the total amount of *Scnn1a* detected by qPCR.

**Figure 5.**
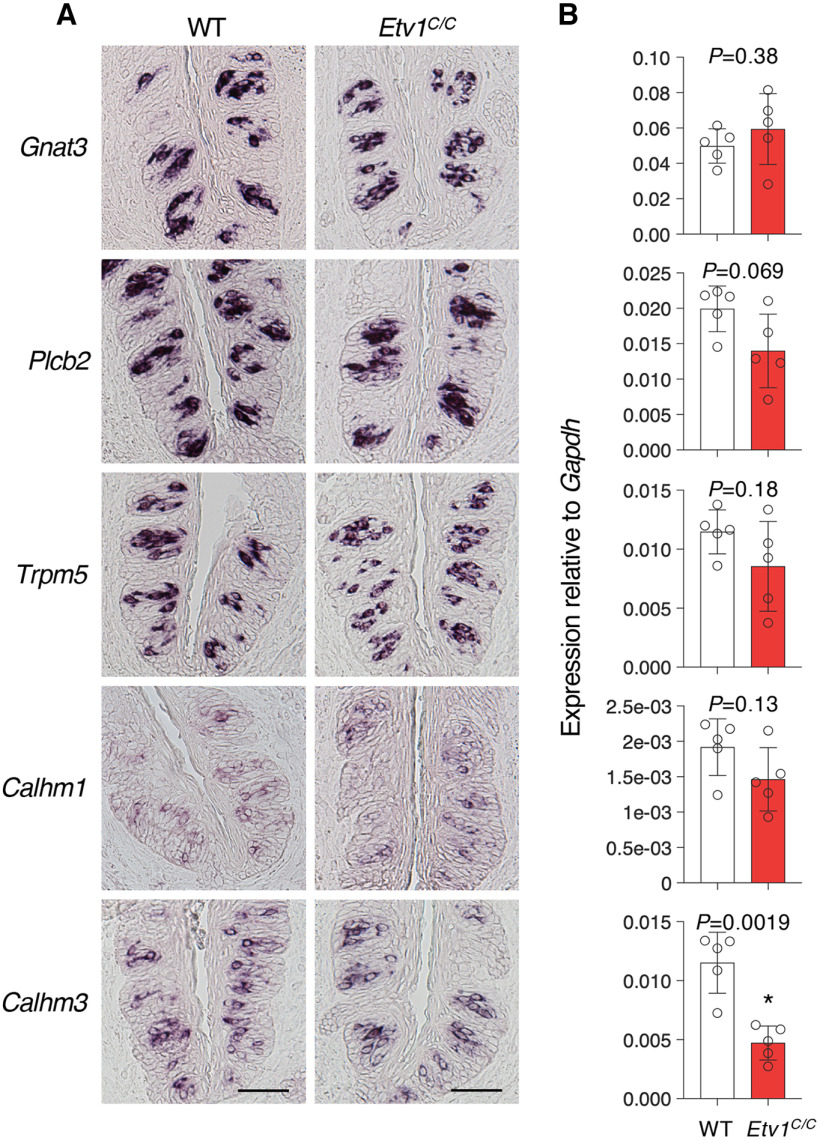
Limited impact of *Etv1* deficiency on expression of taste signaling genes necessary for T1R-mediated tastes. ***A***, Expression of *Gnat3*, *Plcb2*, *Trpm5*, *Calhm1*, and *Calhm3* that are partially co-expressed with *Etv1* in circumvallate papillae of wild-type (WT; left) and *Etv1^C/C^* (right) mice by *in situ* hybridization. *n = *1 for each genotype, for each experiment; 3 sections. Scale bars: 50 μm. ***B***, qPCR analyses in taste buds in circumvallate papillae of WT and *Etv1^C/C^* mice. *n *=* *5 for each genotype. Open circles, individual data; white and red bars are the mean ± SD of WT and *Etv1^C/C^* tissues, respectively. Significance was assessed by Welch’s *t* test. For results in fungiform papillae and soft palate, see Extended Data Figures 5-1 and 5-2, respectively.

Next, we studied the impact of *Etv1* deficiency on the expression of genes encoding the downstream signaling molecules commonly required for sweet, umami, and bitter tastes. *In situ* hybridization analyses showed no obvious differences in the expression of any genes for taste signaling molecules between wild-type and *Etv1^C/C^* mice (Fig. 5*A*; Extended Data Figs. 5-1*A*, 5-2*A*). However, *Calhm3* expression was significantly decreased by *Etv1* deficiency in all gustatory areas examined, and the expression of *Calhm1* and *Plcb2* was also significantly decreased in one or two gustatory area (Fig. 5*B*; Extended Data Figs. 5-1*B*, 5-2*B*). Although it seems to be largely dispensable for the expression of taste signaling molecule genes, Etv1 may participate in their expression.

10.1523/ENEURO.0236-22.2023.f5-1 Extended Data Figure 5-1 Impact of *Etv1* deficiency on expression of taste signaling genes necessary for T1R-mediated tastes in fungiform papillae. ***A***, *Gnat3*, *Plcb2*, *Trpm5*, *Calhm1*, and *Calhm3* are partially co-expressed with *Etv1* in fungiform papillae of wild-type (WT; left) and *Etv1^C/C^* (right) mice by *in situ* hybridization. *n = *1 for each genotype, for each experiment; 9 sections. Scale bars: 25 μm. ***B***, qPCR analyses in taste buds in fungiform papillae of WT and *Etv1^C/C^* mice. *n *=* *5 for each genotype. Open circles, individual data; white and red bars are the mean ± SEM of WT and *Etv1^C/C^* tissues, respectively. Significance was assessed by Welch’s *t* test. Download Figure 5-1, TIF file.

10.1523/ENEURO.0236-22.2023.f5-2 Extended Data Figure 5-2 Impact of *Etv1* deficiency on expression of taste signaling genes necessary for T1R-mediated tastes in palate. ***A***, *Gnat3*, *Plcb2*, *Trpm5*, *Calhm1*, and *Calhm3* are partially co-expressed with *Etv1* in palate of wild-type (WT; left) and *Etv1^C/C^* (right) mice by *in situ* hybridization. *n = *1 for each genotype, for each experiment; 9 sections. Scale bars: 25 μm. ***B***, qPCR analyses in taste buds in palate of WT and *Etv1^C/C^* mice. *n *=* *5 for each genotype. Open circles, individual data; white and red bars are the mean ± SEM of WT and *Etv1^C/C^* tissues, respectively. Significance was assessed by Welch’s *t* test. Download Figure 5-2, TIF file.

**Figure 6.**
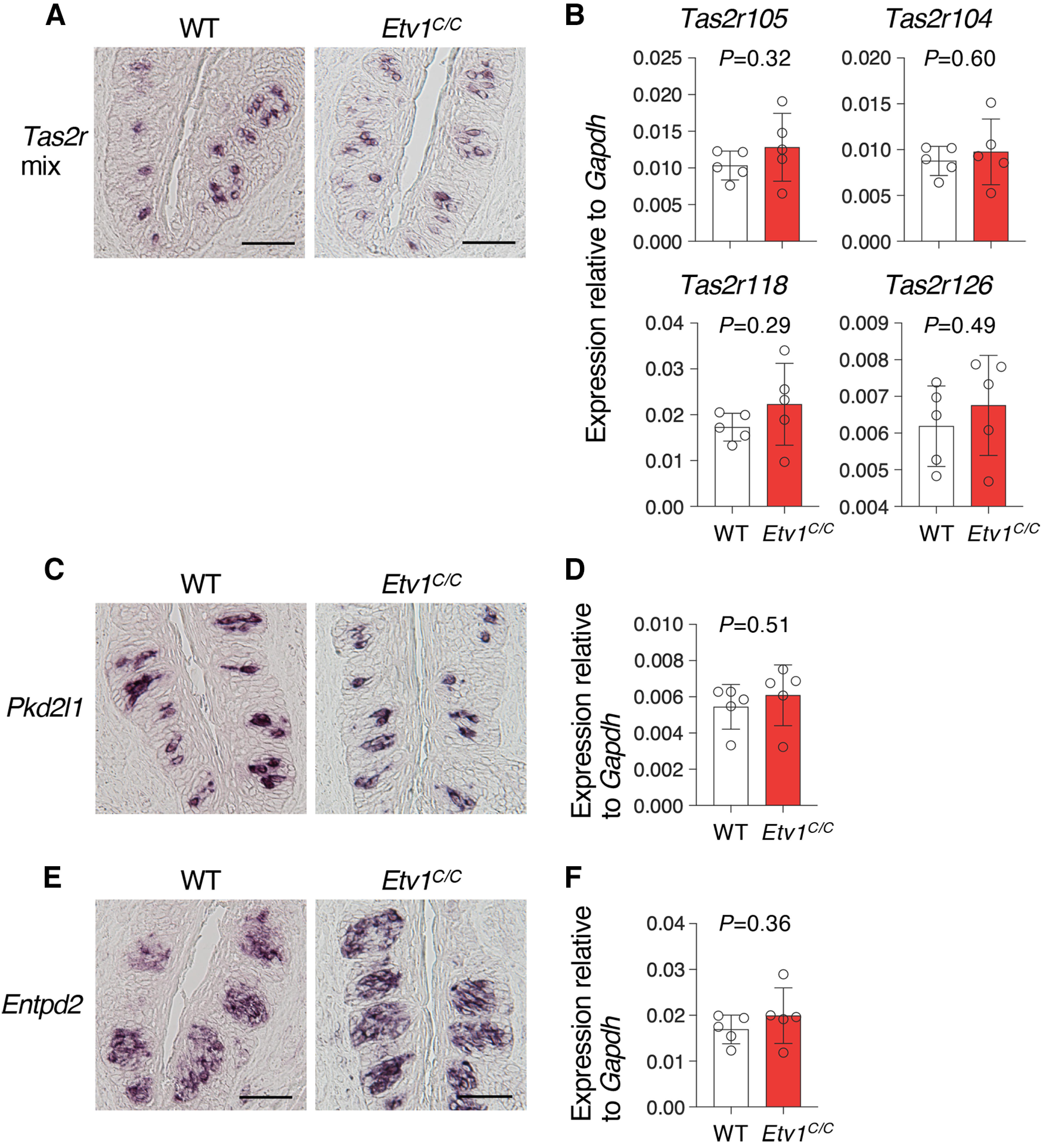
Unaltered expression of genes that are not co-expressed with *Etv1* by *Etv1* deficiency. Expression of T2R genes, *Pkd2l1*, and *Entpd2* was examined in circumvallate papillae of wild-type (WT) and *Etv1^C/C^* mice. ***A***, ***C***, ***E***, *In situ* hybridization. T2R gene expression was examined using a mixture of probes to four T2R genes (*Tas2r104*, *Tas2r105*, *Tas2r118*, and *Tas2r126*). Left, WT. Right, *Etv1^C/C^* mice. For each genotype, *n = *2, with 5 sections for T2R genes; *n *=* *1, with 3 sections for *Pkd2l1* and *Entpd2*. Scale bars: 50 μm. ***B***, ***D***, ***F***, qPCR analyses. T2R genes used for *in situ* hybridization were individually measured (***B***). *n *=* *5 for each genotype. Open circles, individual data; white and red bars are the mean ± SD of WT and *Etv1^C/C^* tissues, respectively. Significance was assessed by Welch’s *t* test. For results in fungiform papillae and soft palate, see Extended Data Figures 6-1 and 6-2, respectively.

We then examined the expression of genes that in wild-type mice are expressed in *Etv1*-negative taste bud cells. Frequency of signals to T2R genes in taste buds of *Etv1^C/C^* mice were comparable to those in wild-type mice by *in situ* hybridization (Fig. 6*A*; Extended Data Figs. 6-1*A*, 6-2*A*). Consistent with this, qPCR analyses showed no significant difference between wild-type and *Etv1^C/C^* mice in CvP and FuP (Fig. 6*B*; Extended Data Fig. 6-1*B*). Intriguingly, however, the expression of three out of four T2R genes examined was significantly increased in palate (Extended Data Fig. 6-2*B*). The expression of *Pkd2l1* and the nonsensory taste bud cell marker *Entpd2* were also unaltered in the taste buds of *Etv1^C/C^* mice compared with wild-type mice (Fig. 6*C–F*; Extended Data Figs. 6-1*C–E*, 6-2*C–E*).

10.1523/ENEURO.0236-22.2023.f6-1 Extended Data Figure 6-1 Unaltered expression of genes that are not co-expressed with *Etv1* in fungiform papillae of *Etv1*-deficient mice. Expression of T2R genes, *Pkd2l1*, and *Entpd2* was examined in fungiform papillae of wild-type (WT) and *Etv1^C/C^* mice. ***A***, ***C***, ***D***, *In situ* hybridization. *Tas2r* mix (***A***) refers to a mixture of probes to four T2R genes (*Tas2r104*, *Tas2r105*, *Tas2r118*, and *Tas2r126*). Left, wild-type (WT). Right, *Etv1^C/C^* mice. *n *=* *1, with 9 sections for WT, and *n *=* *2, with 18 sections for *Etv1^C/C^* for T2R genes; *n *=* *2, with 18 sections for each genotype for *Pkd2l1*; *n *=* *1, with 9 sections for each genotype for *Entpd2*. Scale bars: 25 μm. ***B***, ***E***, qPCR analyses. T2R genes used for *in situ* hybridization were individually measured (***B***), but *Tas2r104*, *Tas2r118*, and *Tas2r126*, as well as *Pkd2l1*, did not show reliable amplification even in WT mice. *n *=* *5 for each genotype. Open circles, individual data; white and red bars are the mean ± SEM of WT and *Etv1^C/C^* tissues, respectively. Significance was assessed by Welch’s *t* test. Download Figure 6-1, TIF file.

10.1523/ENEURO.0236-22.2023.f6-2 Extended Data Figure 6-2 Impact of *Etv1* deficiency on expression of genes that are not co-expressed with *Etv1* in palate. Expression of T2R genes, *Pkd2l1*, and *Entpd2* was examined in palate of wild-type (WT) and *Etv1^C/C^* mice. ***A***, ***C***, ***D***, *In situ* hybridization. *Tas2r* mix (A) refers to a mixture of probes to four T2R genes (*Tas2r104*, *Tas2r105*, *Tas2r118*, and *Tas2r126*). Left, WT. Right, *Etv1^C/C^* mice. *n *=* *1, with 9 sections for WT, and *n *=* *2, with 18 sections for *Etv1^C/C^* for T2R genes; *n *=* *2, with 18 sections for each genotype for *Pkd2l1*; *n *=* *1, with 9 sections for each genotype for *Entpd2*. Scale bars: 25 μm. ***B***, ***E***, qPCR analyses. T2R genes used for *in situ* hybridization were individually measured (***B***). *Pkd2l1* did not show reliable amplification even in WT mice. *n *=* *5 for each genotype. Open circles, individual data; white and red bars are the mean ± SEM of WT and *Etv1^C/C^* tissues, respectively. Significance was assessed by Welch’s *t* test. Download Figure 6-2, TIF file.

Collectively, these results suggest that Etv1 is involved in the differentiation of taste cells by regulating expression of taste receptor and taste signaling molecule genes in sweet, umami, and sodium taste cells.

### Differential impact of *Etv1* deficiency on the composition of CreER^+^ taste cells

**Figure 7.**
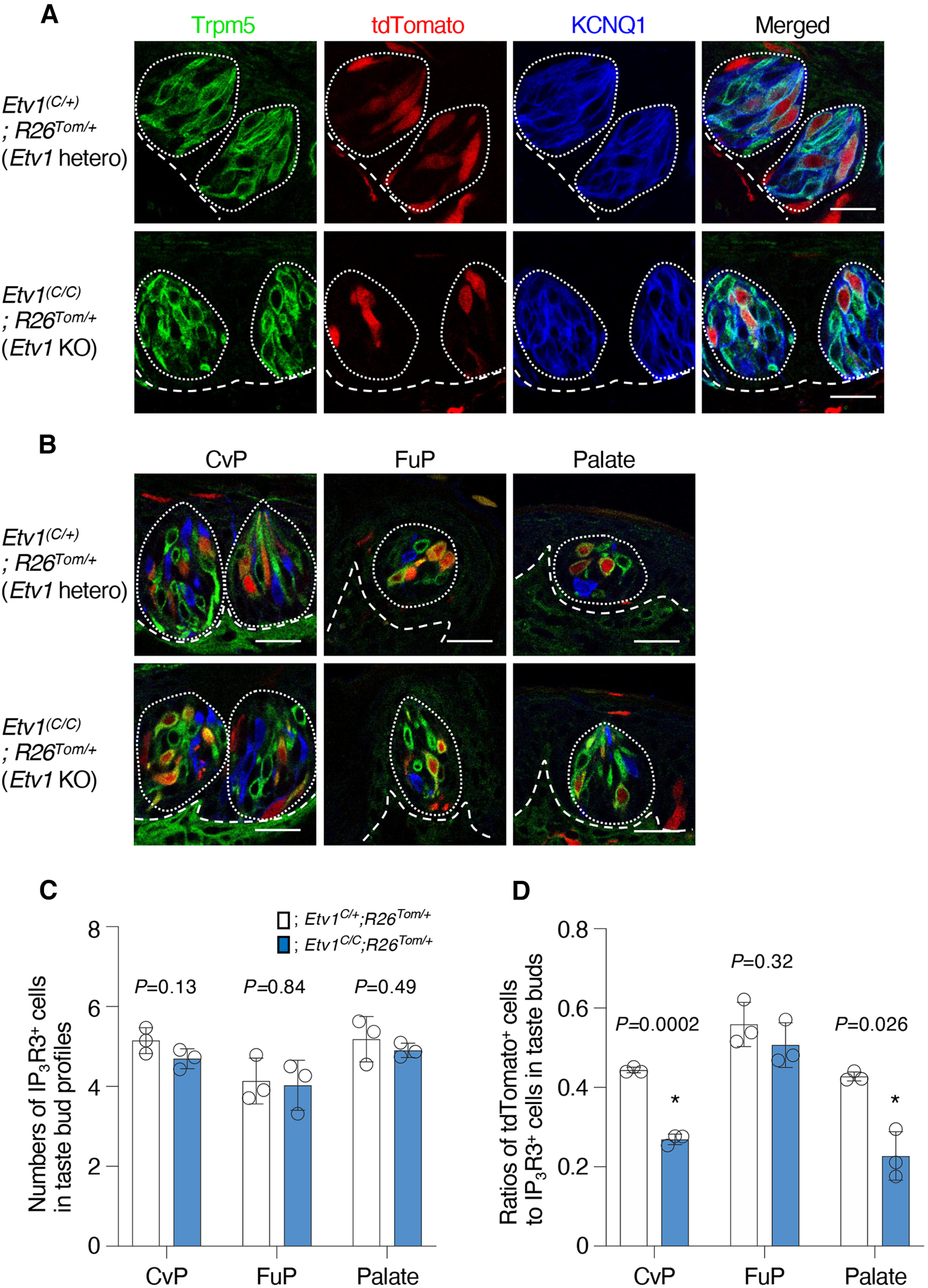
Presence of CreER^+^ taste cells in *Etv1*-deficient taste buds. ***A***, Triple-fluorescence labeling to study the impact of an *Etv1* deficiency on the generation of *Etv1*^+^ taste cells in circumvallate papillae: Trpm5 (green; left) to identify Skn-1a-dependent taste cells, tdTomato (red; left middle) to identify *Etv1*^+^ taste cells, KCNQ1 (blue; right middle) that marks most taste bud cells, and merged images (right) of circumvallate taste buds of *Etv1^C/+^*; *R26^Tom/+^* (top) and *Etv1^C/C^*; *R26^Tom/+^* (bottom) mice. *n = *2, with 6 sections for each experiment. ***B***, Triple-fluorescence images for IP_3_R3 (to identify Skn-1a-dependent taste cells, green) and tdTomato (to identify *Etv1*^+^ taste cells, red), and Ddc (to identify sour taste cells, blue) in taste buds in circumvallate (CvP; left) and fungiform (FuP; center) papillae and palate (right) of *Etv1^C/+^*; *R26^Tom/+^* (top) and *Etv1^C/C^*; *R26^Tom/+^* (bottom) mice (*n = *3 for each genotype). Taste buds and basal epithelial-mesenchymal boundaries are indicated by broken lines. Scale bars: 25 μm. ***C***, ***D***, Quantitative analyses of taste cell populations. The number of Skn-1a-dependent taste cells (***C***) and ratios of tdTomato^+^ cells to Skn-1a-dependent taste cells (***D***) in single taste bud profiles in the CvP, FuP, and palate were compared between *Etv1^C/+^*; *R26^Tom/+^* (white bar) and *Etv1^C/C^*; *R26^Tom/+^* (blue bar) mice. Data are expressed as the mean ± SD. Significance was assessed by Welch’s *t* test. For the populations of total taste bud cells and sour taste cells in single taste bud profiles, see Extended Data Figure 7-1.

The residual expression of T1R genes in the *Etv1*-deficient mice indicates that Etv1 is not indispensable for the generation of taste cells. To evaluate whether *Etv1* deficiency influences *Etv1*^+^ taste cell populations, we generated *Etv1^C/C^*; *R26^Tom/+^* mice (in which tdTomato expression is activated in *Etv1*^+^ cells by tamoxifen injection) and examined whether tdTomato expression was observed in taste buds. In all gustatory areas examined, tdTomato expression was observed in a subset of taste bud cells of *Etv1^C/C^*; *R26^Tom/+^* mice (Fig. 7*A*,*B*). Presence of tdTomato in taste buds of *Etv1^C/C^*; *R26^Tom/+^* mice suggests that *Etv1* deficiency is not crucial for generation or maintenance of taste cells (Fig. 7*A*,*B*). We then identified Skn-1a-dependent taste cells using specific marker IP_3_R3; distinguished *Etv1*^+^ and *Etv1*^–^ taste cells in Skn-1a-dependent cells as tdTomato^+^ and tdTomato^–^ cells, respectively; identified sour taste cells by the expression of sour taste cell marker Ddc; and calculated their number in single taste buds. The population of tdTomato^+^ cells was significantly decreased to about half in CvP and palate of *Etv1^C/C^*; *R26^Tom/+^* mice compared with that in the *Etv1^C/+^*; *R26^Tom/+^* heterozygous control mice, while the populations of KCNQ1^+^, Ddc^+^, and IP_3_R3^+^ cells were unaltered in any gustatory areas (Fig. 7*C*,*D*; Extended Data Fig. 7-1). Intriguingly, no significant difference in the population of tdTomato^+^ cells was observed in FuP between *Etv1^C/C^*; *R26^Tom/+^* and *Etv1^C/+^*; *R26^Tom/+^* heterozygous control mice (Fig. 7*B*,*C*). These results suggest that Etv1 is involved in generation and/or maintenance of taste cells. In CvP, the impact on the population of tdTomato^+^ cells by *Etv1* deficiency is less pronounced than that on gene expression, and the expression of T2R genes is not increased (Fig. 6). Although we cannot completely preclude the increase of bitter taste cells and the involvement of Etv1 in cell fate choice toward sweet, umami, or sodium taste cells over bitter taste cells, the decrease of tdTomato^+^ cells (and thus the increase of tdTomato^–^ cells) seems to be simply explained by the decrease of CreER expression from *Etv1* gene: Etv1 may be involved in the positive-feedback regulation of *Etv1* gene expression in taste cells.

10.1523/ENEURO.0236-22.2023.f7-1 Extended Data Figure 7-1 Little impact by *Etv1* deficiency on taste bud cell populations. Numbers of KCNQ1^+^ (***A***) and Ddc^+^ (***B***) cells in single taste bud profiles in the CvP, FuP, and palate were compared between *Etv1^C/+^*; *R26^Tom/+^* (white bar) and *Etv1^C/C^*; *R26^Tom/+^* (blue bar) mice. Data are expressed as the mean ± SEM. Significance was assessed by Welch’s *t* test. Download Figure 7-1, TIF file.

### *Etv1* expression in the cranial sensory neurons

**Figure 8.**
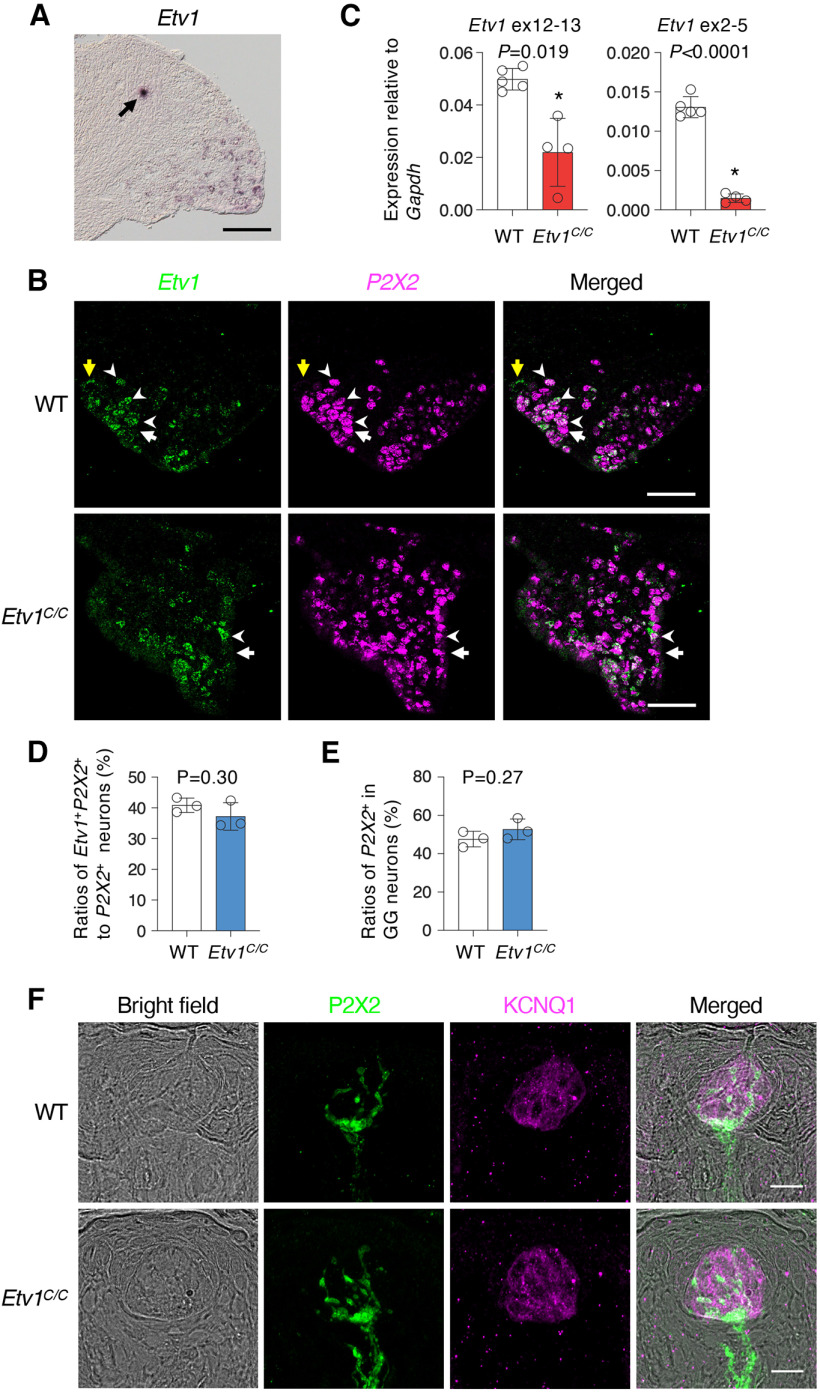
Expression of *Etv1*-related transcripts in geniculate ganglion neurons. ***A***, *In situ* hybridization to show the expression of *Etv1* and/or *Etv1*-related transcripts in geniculate ganglia of wild-type mice. Black arrow indicates nonspecific staining in the trunk of facial nerve. Scale bar: 100 μm. ***B***, Double-label *in situ* hybridization to study expression of *P2x2* in *Etv1*^+^ neurons: *Etv1* (green; left), *P2x2* (magenta; middle), and merged (right) images of geniculate ganglia in wild-type (WT; top) and *Etv1^C/C^* (bottom) mice. White arrowheads, *Etv1*^+^*P2x2*^+^ neurons; white arrows, *Etv1*^−^*P2x2*^+^ neurons; and yellow arrows, *Etv1*^+^*P2x2*^−^ neurons. Scale bars: 100 μm. ***C***, qPCR analyses to detect the expression of *Etv1* and/or *Etv1*-related transcripts in geniculate ganglia. Primers used are sets N and C (for details, see Fig. 2*C*; Table 2). *n *=* *5 for WT; *n *=* *4 for *Etv1^C/C^* mice. ***D***, ***E***, Ratio of *Etv1*^+^*P2x2*^+^ neurons to *P2x2*^+^ neurons (***D***) and ratio of *P2x2*^+^ neurons to total neurons (***E***) in geniculate ganglia in WT and *Etv1^C/C^* mice. *n = *3 for each genotype. Data are expressed as the mean ± SD. Significance was assessed by Welch’s *t* test. ***F***, P2X2-immunoreactive fiber projection into taste buds in fungiform papillae: bright field (left), P2X2 (green; left middle), KCNQ1 (magenta; right middle), and merged images (right) of fungiform papillae of WT (top) and *Etv1^C/C^* (bottom) mice. Fluorescent images of P2X2^+^ signals were merged images of serial z-stacks. *n *=* *2, with 3–4 sections for each genotype. Scale bars: 20 μm. For results in circumvallate papillae and soft palate, see Extended Data Figure 8-1.

Axotomy of gustatory nerves results in degeneration of taste cells (Guth, 1957; Kennedy, 1972; John et al., 2003; Guagliardo and Hill, 2007; Takeda et al., 2013). To evaluate whether the decrease of *Etv1*^+^ taste cells in *Etv1^C/C^* mice is a cell-autonomous effect or a noncell-autonomous effect caused by the loss of gustatory neurons, we interrogated whether *Etv1* is expressed in sensory neurons in geniculate ganglia (GG) and, if it is expressed, whether *Etv1* is required for their generation and/or innervation. *In situ* hybridization yielded moderate signals in GG neurons in wild-type mice (Fig. 8*A*), predominantly in a minor subset of gustatory neurons expressing *P2x2* (Finger et al., 2005; Dvoryanchikov et al., 2017; Fig. 8*B*). Although qPCR showed little expression of *Etv1*, *Etv1*-derived transcripts putatively encoding Etv1 isoforms were amplified in GG in *Etv1^C/C^* mice (Fig. 8*C*). Consistently, signals of *Etv1*-derived transcripts were observed in *P2x2*-expressing GG neurons in *Etv1^C/C^* mice by *in situ* hybridization (Fig. 8*B*). Importantly, the ratios of *Etv1*^+^*P2x2*^+^ neurons to *P2x2*^+^ neurons and *P2x2*^+^ neurons to all GG neurons are comparable between *Etv1^C/C^* and wild-type mice (Fig. 8*D*,*E*). P2X2^+^ fibers also comparably projected to taste buds, but not to surrounding epithelium devoid of anti-KCNQ1 antibody immunoreactivity, from mesenchymal tissues in both wild-type and *Etv1^C/C^* mice (Fig. 8*F*; Extended Data Fig. 8-1). Etv1 and its isoform(s) appear to be dispensable for the generation and survival of putative gustatory neurons.

10.1523/ENEURO.0236-22.2023.f8-1 Extended Data Figure 8-1 No obvious impact on P2X2-immunoreactive fiber projection into taste buds by Etv1 deficiency. Peripheral sensory fibers were immunohistochemically visualized using anti-P2X2 antibodies, and serial z-stacks were merged (green; left middle). Taste buds were labeled by KCNQ1 immunoreactivity (magenta; right middle) in the circumvallate papillae (***A***) and soft palate (***B***) of wild-type (WT; top) and *Etv1^C/C^* (bottom) mice. Fluorescence images together with confocal transmitted light images (left) were merged (right). P2X2^+^ fiber projection was predominantly observed in taste buds. *n *=* *2, with 3–4 sections for each genotype. Scale bars: 20 μm. Download Figure 8-1, TIF file.

### Taste responsiveness of gustatory nerves by *Etv1* deficiency

**Figure 9.**
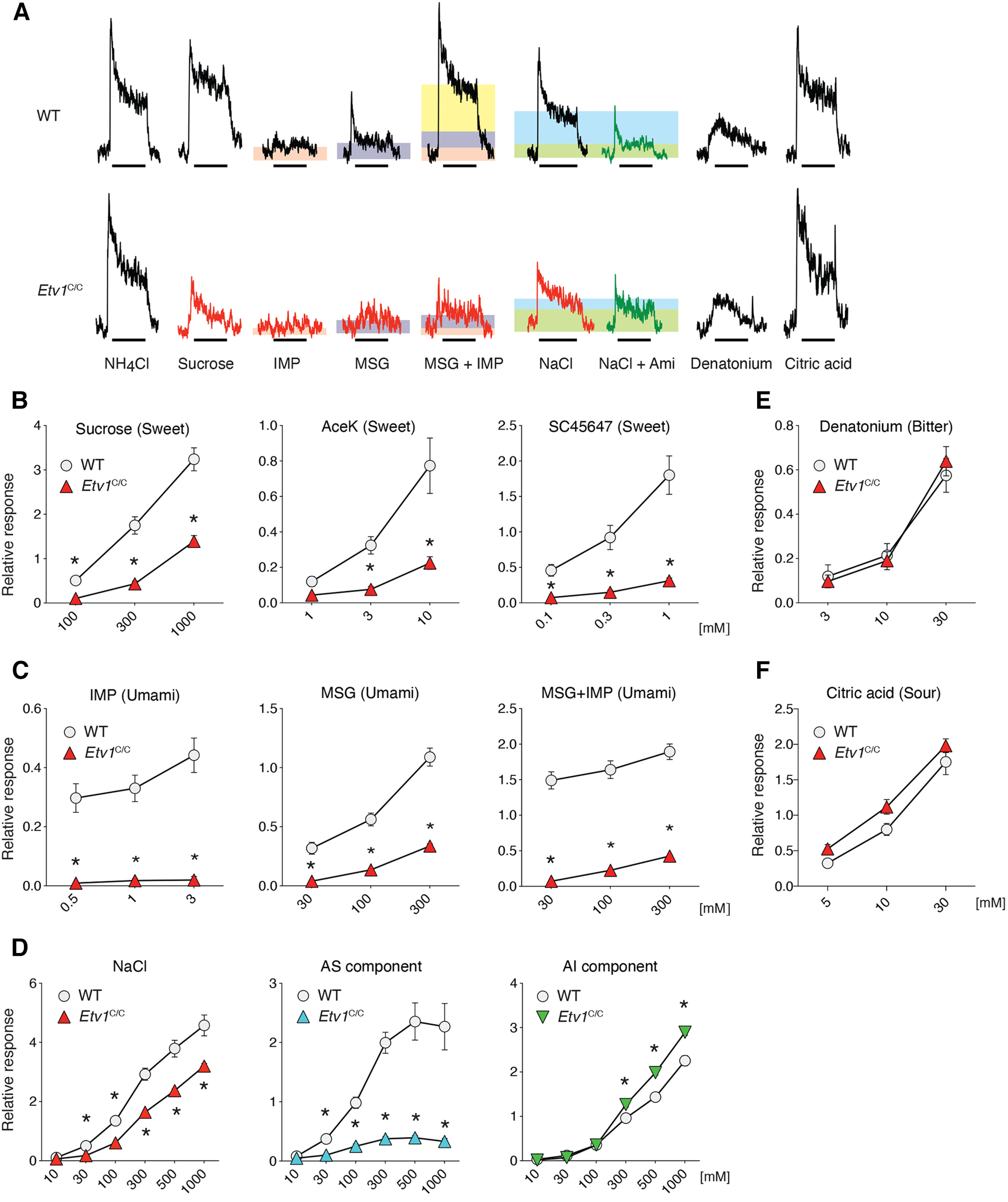
Etv1 deficiency in taste cells impairs neural responses to sweet, umami, and sodium tastes. ***A***, Representative charts of chorda tympani nerve responses of wild-type (WT; top) and *Etv1^C/C^* (bottom) mice to taste solutions: 100 mm NH_4_Cl, 300 mm sucrose, 0.5 mm inositol monophosphate (IMP), 100 mm monosodium glutamate (MSG), 100 mm MSG with 0.5 mm IMP, 100 mm sodium chloride (NaCl), 100 mm NaCl with 100 μm amiloride (Ami), 30 mm denatonium, and 10 mm citric acid. Responses of 0.5 mm IMP (orange) and 100 mm MSG (purple) are depicted as shaded rectangles to clarify the synergy (yellow) in the response of 100 mm MSG with 0.5 mm IMP. Responses to NaCl are depicted as shaded rectangles for amiloride-sensitive (blue) and amiloride-insensitive (green) components. The bars under the traces show the duration (30 s) of the taste stimulus. ***B***, Whole chorda tympani nerve responses of WT and *Etv1^C/C^* mice to sweet taste solutions. Left, Sucrose. Middle, Acesulfame K (AceK). Right, SC45647. ***C***, Whole chorda tympani nerve responses of wild-type and *Etv1^C/C^* mice to umami taste solutions. Left, IMP. Middle, MSG. Right, MSG with 0.5 mm IMP. ***D***, Whole chorda tympani nerve responses of WT and *Etv1^C/C^* mice to NaCl solutions. Amiloride-sensitive salt responses (AS component; middle) are measured by subtracting the amiloride-insensitive response (AI component; right) from the whole salt response (left). ***E***, ***F***, Whole chorda tympani nerve responses of WT (circles) and *Etv1^C/C^* (triangles) mice to bitter (***E***) and sour (***F***) taste solutions that are received by receptors other than T1Rs (*n = *8 for each genotype). Significance was assessed by repeated-measures two-way ANOVA and the Sidak’s multiple comparison test. *Significantly different (*p *<* *0.05) responses between genotypes. Data are expressed as the mean ± SEM; where error bars are not visible, they are smaller than the symbol depicting the mean. See Table 3 for statistical values.

Lastly, we examined electrophysiological responses to various taste substances from chorda tympani nerves that innervate taste cells in the FuP (Fig. 9*A*). Chorda tympani nerve responses to sucrose were significantly reduced but still observed in the *Etv1^C/C^* mice, and a greater reduction in responses to artificial sweeteners was observed (Fig. 9*B*; Table 3). *Etv1^C/C^* mice exhibited decreased chorda tympani nerve responses to inosine monophosphate and monosodium glutamate, but their synergistic effects were not observed: the responses of MSG+IMP are comparable to the sums of individual responses of MSG and IMP in *Etv1^C/C^* mice (Fig. 9*C*; Table 3). *Etv1^C/C^* mice showed severely reduced chorda tympani nerve responses to sodium chloride (NaCl) and no amiloride-sensitive components of the nerve responses to NaCl, while the amiloride-insensitive components remained almost normal (Fig. 9*D*; Table 3). We observed no significant changes in chorda tympani nerve responses to chemicals with a bitter or sour taste in the *Etv1^C/C^* mice (Fig. 9*E*,*F*; Table 3). These results of chorda tympani nerve recordings are well consistent with those of gene expression analyses in FuP. Together with the unaltered populations of putative gustatory neurons in GG and *Etv1*^+^ taste cells in FuP, it is likely that gustatory neurons innervate taste cells in FuP in *Etv1^C/C^* mice and that the altered gene expression in *Etv1*^+^ taste cells in *Etv1^C/C^* mice is a cell-autonomous effect of *Etv1* deficiency in taste cells. The decreased responses of chorda tympani nerves to sweet, umami, and sodium taste substances in *Etv1^C/C^* mice should thus be functional consequences of the diminished expression of T1R genes and *Scnn1a* because of *Etv1* deficiency in taste cells.

**Table 3:** Summary of statistical analyses of chorda tympani responses of wild-type (WT) and *Etv1^C/C^* mice to taste compounds

Quality	Compound (component)	Genotype (WT vs *Etv1^C/C^*)		Concentration		Interaction		Concentration differing significantly with *p* value^*^
Sweet	Sucrose	*F*_(1,14)_ = 43.3	*p* < 0.0001	*F*_(1.274,17.83)_ = 249.2	*p* < 0.0001	*F*_(2,28)_ = 32.5	*p* < 0.0001	100 mm (*p* = 0.0023)
								300 mm (*p* = 0.0005)
								1000 mm (*p* = 0.0002)
	Acesulfame K	*F*_(1,14)_ = 15.9	*p* = 0.0014	*F*_(1.072,15.01)_ = 27.26	*p* < 0.0001	*F*_(2,28)_ = 8.5	*p* = 0.0013	3 mm (*p* = 0.0036)
								10 mm (*p* = 0.028)
	SC45647	*F*_(1,14)_ = 26.0	*p* = 0.0002	*F*_(1.269,17.76)_ = 58.94	*p* < 0.0001	*F*_(2,44)_ = 52.3	*p* < 0.0001	0.1 mm (*p* = 0.0064)
								0.3 mm (*p* = 0.0075)
								1 mm (*p* = 0.0025)
Umami	Inosine	*F*_(1,14)_ = 48.9	*p* < 0.0001	*F*_(1.759,24.63)_ = 15.34	*p* < 0.0001	*F*_(2,28)_ = 12.1	*p* = 0.0002	0.5 mm (*p* = 0.0016)
	monophosphate (IMP)							1 mm (*p* = 0.0005)
								3 mm (*p* = 0.0004)
	Monosodium	*F*_(1,14)_ = 68.5	*p* < 0.0001	*F*_(1.669,23.37)_ = 408.9	*p* < 0.0001	*F*_(2,28)_ = 80.6	*p* < 0.0001	30 mm (*p* = 0.0020)
	glutamate (MSG)							100 mm (*p* = 0.0002)
								300 mm (*p* < 0.0001)
	MSG+IMP^*a*^	*F*_(1,14)_ = 172.0	*p* < 0.0001	*F*_(1.340,18.76)_ = 43.54	*p* < 0.0001	*F*_(2,28)_ = 0.28	*p* = 0.76	30 mm (*p* < 0.0001)
								100 mm (*p* < 0.0001)
								300 mm (*p* < 0.0001)
Salty^*b*^	NaCl	*F*_(1,14)_ = 33.3	*p* < 0.0001	*F*_(1.881,26.33)_ = 347.0	*p* < 0.0001	*F*_(5,70)_ = 12.4	*p* < 0.0001	30 mm (*p* = 0.0013)
								100 mm (*p* = 0.0007)
								300 mm (*p* = 0.0022)
								500 mm (*p* = 0.0081)
								1000 mm (*p* = 0.033)
	(AS salt)	*F*_(1,14)_ = 53.6	*p* < 0.0001	*F*_(1.704,23.85)_ = 38.07	*p* < 0.0001	*F*_(5,70)_ = 21.7	*p* < 0.0001	30 mm (*p* = 0.0059)
								100 mm (*p* = 0.0003)
								300 mm (*p* = 0.0002)
								500 mm (*p* = 0.0024)
								1000 mm (*p* = 0.0095)
	(AI salt)	*F*_(1,14)_ = 20.3	*p* = 0.0005	*F*_(1.637,22.92)_ = 1060	*p* < 0.0001	*F*_(5,70)_ = 23.9	*p* < 0.0001	300 mm (*p* = 0.0029)
								500 mm (*p* = 0.0006)
								1000 mm (*p* = 0.0026)
Bitter	Denatonium	*F*_(1,14)_ = 0.0072	*p* = 0.93	*F*_(1.224,17.13)_ = 119.8	*p* < 0.0001	*F*_(2,28)_ = 1.1	*p* = 0.35	
Sour	Citric acid	*F*_(1,14)_ = 3.9	*p* = 0.068	*F*_(1.368,19.15)_ = 230.8	*p* < 0.0001	*F*_(2,28)_ = 0.4	*p* = 0.67	

* *p* < 0.05 according to *post hoc* Sidak’s multiple comparison test.

a MSG solutions with 0.5 mm IMP.

b Responses to NaCl were recorded in the absence and presence of amiloride so that amiloride-sensitive (AS) salt responses were calculated by subtracting amiloride-insensitive (AI) responses from whole NaCl responses.

## Discussion

Continuous normal turnover of taste cells underlies homeostasis of the sense of taste. Single stem cells can give rise to any type of taste bud cell (Ren et al., 2014), but the molecular mechanisms that generate specific individual taste bud cells remain poorly understood. This study revealed that *Etv1* participates in the differentiation of Skn-1a-dependent sweet, umami, and sodium taste cells by regulating the expression of taste receptor genes in them. Etv1 may be a part of the mechanism that, among Skn-1a-dependent cells, distinguishes sweet, umami, and sodium taste cells from bitter taste cells.

### Differentiation of taste cells

*Etv1* is expressed in sweet, umami, and sodium taste cells, and its deficiency impaired expression of genes encoding taste receptors and intracellular signal transduction molecules necessary for sweet, umami, and sodium tastes. Therefore, Etv1 appears to be involved in the differentiation of these taste cells in multiple ways. However, *Etv1* deficiency does not completely abolish the expression of taste receptor genes. There may be other molecules that are functionally redundant to Etv1 and may have partially compensated for the loss of Etv1 in taste cells in *Etv1^C/C^* mice; for example, Etv6 is expressed in *Tas1r3*^+^ taste cells (Lee et al., 2017; Sukumaran et al., 2017). We presume that transcription factors specific to sweet, umami, and sodium taste cells, respectively, are expressed to characterize individual taste cell subsets and may compensate for Etv1 function to some extent in *Etv1^C/C^* mice, which explains the differential impacts of *Etv1* deficiency on gene expression. However, this hypothesis cannot fully explain regional differences in gene expression in specific taste cell subsets (e.g., *Tas1r2* expression in sweet taste cells in FuP and palate but not in CvP) or the effects on the composition of Skn-1a-dependent taste cells by *Etv1* deficiency. There may be more varieties of transcription factors in taste cells and more molecular mechanisms to generate such varieties than we understand today.

Expression of *Etv1* and *Eya1* seems to be reversely correlated in *Skn-1a*^+^ cells in taste buds: *Eya1* is expressed in the putative precursor and bitter taste cells (Ohmoto et al., 2021), while *Etv1* is expressed in the remaining *Skn-1a*^+^ cell types. In addition to its functions as a positive regulator of gene expression, Etv1 may negatively regulate T2R genes as a repressor in sweet, umami, and sodium taste cells. Partially consistent with this possibility, the expression of three out of four T2R genes examined was increased only in palate of the *Etv1^C/C^* mice. This regional discrepancy may be explained by the different degrees of compensation by distinct, yet-to-be-identified transcription regulators that cooperate with Etv1 in sweet, umami, and sodium taste cells, as discussed above. Because these taste cells differ in their spatial distributions (Hoon et al., 1999; Chandrashekar et al., 2010; Ohmoto et al., 2020a), regional differences in impacts of Etv1 deficiency are perhaps unsurprising.

Many genes that encode transcription factors involved in the regulation of gene expression are expressed in taste buds (Hevezi et al., 2009; Lee et al., 2017; Sukumaran et al., 2017). Some of them may play crucial roles in specifying respective taste cells and maintaining their specific identities. Like Sox2 in stem cells and Skn-1a in precursor cells, several factors would act as selectors at several crossroads in the differentiation process of taste bud cells. It would be very interesting to identify which transcription factor genes are expressed in which taste cells by single-cell RNAseq and to analyze which genes’ 5’-upstream regions are accessible for transcription machinery in individual taste cells by single-cell ATAC-seq.

### Tissue-specific or cell-specific isoforms of Etv1

The *Etv1* mutant strain used in this study, *Etv1^C/C^*, is designed to encode a tamoxifen-inducible Cre recombinase CreER instead of Etv1, analogous to another *Etv1* mutant strain, *Etv1^nlslacZ/nlslacZ^*, which is designed to encode a nuclear-localized β-galactosidase (Extended Data Fig. 2-1*A*; Arber et al., 2000; Taniguchi et al., 2011). Therefore, these two strains were expected to exhibit similar phenotypes: impaired locomotion because of deficits in proprioceptive neuronal circuits, and a short life span (up to several weeks), as observed in *Etv1^nlslacZ/nlslacZ^* mice, in which Etv1-like immunoreactivity was remarkably reduced in DRG neurons (Arber et al., 2000). However, *Etv1^C/C^* mice exhibit no overt abnormality in locomotion, and this strain can be maintained as homozygous. More notable, Etv1-like immunoreactivity in DRG in *Etv1^C/C^* mice is observed in the proprioceptive sensory neurons, comparable to wild-type mice (Fig. 2). Also, qualitative and quantitative PCR analyses demonstrated no significant change of *Etv1*-derived transcript in DRG in *Etv1^C/C^* mice, consistent with qualitative observations by *in situ* hybridization analyses. However, no cDNA fragment was amplified from N-terminal coding exons, probably because transcription from the first exon of *Etv1* is stopped by the insertion of CreERT2 in the second exon (Extended Data Fig. 2-1*C*). The fact that *Etv1*-v2 detected as a major transcript in DRG by 5’-RACE starts its transcription from the sequence in the fourth intron of *Etv1* (Extended Data Fig. 2-1*B*,*C*) can account for little, if any, impact on its expression by CreERT2 insertion. The discrepancy of *Etv1*-v2 expression level between *Etv1^nlslacZ/nlslacZ^* and *Etv1^C/C^* mice may be attributed to the remaining *pgk-Neo* sequence in the *Etv1* locus of *Etv1^nlslacZ/nlslacZ^* mice (Extended Data Fig. 2-1*A*), as reported in other alleles (Fernex et al., 1997; Nagy et al., 1998; Kist et al., 2005; Joo et al., 2007; Andersen et al., 2014).

Intriguingly, *Etv1*-derived transcripts are also expressed in putative gustatory neurons in GG in wild-type and *Etv1^C/C^* mice, but unlike in proprioceptive neurons in DRG and in taste cells, in GG in *Etv1^C/C^* mice the expression of *Etv1*-derived transcripts is significantly decreased but not lost. Species and amounts of Etv1 isoforms seem to vary among cells and/or tissues. Identifying isoforms and examining the phenotypes in Etv1^+^ cells in *Etv1^C/C^* mice may provide insights into structure-function relationships of Etv1. For example, because Etv1 is absent in DRG in *Etv1^C/C^* mice, which do not display impaired locomotion or proprioception, such phenotypes observed in *Etv1^nlslacZ/nlslacZ^* and *Etv1^ETS/ETS^* mice can be attributable to the loss of Etv1 isoform b encoded by *Etv1*-v2 in proprioceptive neurons but not to the loss of Etv1, although they may be functionally redundant. It is suggested that Etv1 is involved in different processes in multiple malignant tissues, such as carcinogenesis of prostate cancer, cell survival of gastrointestinal stromal tumor, and metastasis of pancreatic cancer (Tomlins et al., 2005; Chi et al., 2010; Heeg et al., 2016). It may be not Etv1 but an isoform that is involved in those pathologic conditions. *Etv1^C/C^* mice, together with *Etv1^nlslacZ/nlslacZ^* and *Etv1^EST/EST^* mice, will be very valuable models to understand how Etv1 and its isoforms are involved in those processes in cancer studies, in addition to studying their roles in normal adult tissues.

## Synthesis

Reviewing Editor: Christina Zelano, Northwestern University - Chicago Decisions are customarily a result of the Reviewing Editor and the peer reviewers coming together and discussing their recommendations until a consensus is reached. When revisions are invited, a fact-based synthesis statement explaining their decision and outlining what is needed to prepare a revision will be listed below. The following reviewer(s) agreed to reveal their identity: Sue Kinnamon.

The reviewers and I agreed that this study is interesting and significant, and addresses an important question about differentiation of taste cells. However, significant concerns were raised by both reviewers that need to be addressed. Please see the detailed comments of the reviewers below, all of which should be addressed on revision:

Reviewer 1:

In this article, the authors characterized specific types of taste cells using Etv1CreERT2 mice as a null mutant model in taste cells. The topic is interesting and of significance. However, some major and minor concerns exist.

1. Some of the conclusions were not supported by the data analyses, which raise major concerns. For examples,

(1) Based on the finding “Etv1 is expressed in sweet, umami, and sodium taste cells, and its deficiency impaired expression of genes encoding taste receptors and intracellular signal transduction molecules necessary for sweet, umami, and sodium tastes.” the authors claimed that “Therefore, Etv1 appears to be involved in the functional differentiation of these taste cells.” First of all, the meaning of “functional differentiation” was not clarified. Secondly, to claim that Etv1 is involved in the differentiation of specific type(s) of taste cells for particular functions, analyses of the total number of taste buds, number of taste bud cells per bud, and proportions of each type of taste cells relative to total taste bud cells would be important. Current findings lead to a reasonable thought that as a transcription factor Etv1 regulates the expression of certain taste receptor genes.

(2) Based on the observations that “Etv1 deficiency does not completely abolish the expression of taste receptor genes in them.”, then the authors speculated that “There may be other molecules that are functionally redundant to Etv1 to some extent, and they may have partially compensated for the loss of Etv1 in taste cells of the Etv1CreERT2 mice.” The tissue collections were performed one day after the tamoxifen administration (5 consecutive days). The experimental design does not exclude the possibility that some of the Etv1+ taste cells are long-living or Etv1 is not involved in the survival of these taste cells. A longitudinal observation would be helpful.

(3) The authors need to be careful to say that “numbers of taste bud profiles exhibiting signals were significantly decreased in the taste buds in FuP and palate of Etv1CreERT2 mice when signal was developed for a shorter time (Fig. 3-1C, D), indicating the decrease in Tas1r3 expression by Etv1 deficiency.” The qRT-PCR does not support it.

2. In addition to analyze the neurons in gustatory ganglia, evaluation of gustatory nerve fibers (e.g., P2X2 immunohistochemistry) in the taste papillae and palate would be helpful for ruling out the possibility of involvement of sensory nerve defects in the reduced sweet, umami and salty taste.

3. In “Methods”, the semiquantitative analyses of frequencies of expression of T1R and Scnn1a genes were confusing and need to be clarified. As described, “frequencies of expression of Scnn1a in Skn-1a+ cells were semiquantitatively analyzed by calculating the ratios of taste bud profiles that harbored at least one cell co-expressing Skn-1a and Scnn1a to all taste bud profiles using every 8 and 10 sections of palate and FuP, respectively, of Etv1+/+ (wild-type control, n = 3) and Etv1CreERT2 (Etv1-deficient, n=3) mice.”. Did the authors mean that taste bud profiles that did not harbor a cell co-expressing Skn-1a were excluded?

4. In “Methods”, “the chorda tympani nerve was dissected free from its junction with the lingual nerve near the tympanic bulla; then the nerve was cut and the central part was placed on a platinum wire recording electrode.” It is not clear how the central part could be used for recording the taste bud stimulation-induced nerve activities after the cut (disconnection with sensory organs).

5. Full names of terms would be helpful when their abbreviations were first introduced, e.g., ETC.

Reviewer 2:

General Comments:

This manuscript reports two major findings: (1) that the transcription factor Etv1 is expressed in taste cells that transduce sweet, umami, and amiloride-sensitive sodium taste and helps push their differentiation toward these appetitive qualities, and (2) that different Etv1 transcripts are expressed differentially in different cell types in mice. These are important findings since little is currently known about factors that regulate taste cell differentiation and turnover. This manuscript is a timely and important contribution to the field and should definitely be published. Unfortunately, their data presentation and poor writing makes it difficult to access the information and make sense of some of their experiments.

First, there are several different lines of transgenic mice utilized and they are designated differently in different parts of the manuscript; further many have confusing names that appear similar unless one carefully examines superscripts. The authors should include a table in the methods that indicates the official strain name of each mouse line used, including lines generated in-house by breeding; their given designation in the text and figures (i.e., Etv1 KO, Etv1 Het, etc.), the phenotype of the line; and, in knockouts tissues where Etv1 is still expressed. It is very hard to see the tiny pluses that are used to designate some of the lines from each other and they are not used consistently in some of the figures.

Second, many passages are difficult to interpret because of poor English usage and grammar. In addition, some of the figures are referenced incorrectly in the text. Many of these problems are listed in specific comments below but this list should not be considered definitive. The entire manuscript needs to be seen and corrected for English usage before publication.

Third, there are some generalizations in the manuscript that, although referenced, are not universally accepted in the taste field. The alternative studies should also be referenced. For example, it is controversial whether all the taste cells are specifically tuned to a single quality, especially at higher concentrations of tastants (e.g., Wu et al., 2015; Dutta et al 2022). The authors are selecting the studies to reference that support their hypothesis of the labeled line theory and ignoring the references that come to alternate conclusions. Similarly, the authors avoid using the common nomenclature in the field regarding cell types, e.g. Type II, Type III and rely entirely on a nomenclature referring to stimulus responsiveness, which as mentioned above, is specious.

Specific comments:

1. The opening lines of the abstract direct the reader’s attention to cell turnover, which is not the focus of this paper. The abstract should start in a more direct fashion. Lines 44-46 are rather convoluted and should be rephrased more clearly. One possibility, “Unlike some complete Etv1-knockout lines, the Etv1-CreERT2 mutant mouse continues to express Etv1 in dorsal root ganglia and some other areas but nonetheless lacks Etv1 expression in taste buds”.

2. The Significance statement starts much too diffusely.

3. Line 86: Stone et al would not seem to be the correct reference for the point being made.

4. Line 92: This paragraph does not flow logically from the preceding.

5. Line 100: Instead of this paragraph summarizing the results, the authors need to discuss the various Etv1 KO mice and what is known already about them. The text that appears at line 291 would be a good start. How do the constructs for the 2 KO lines differ? Is the transgene inserted in the same locus and the same way? Showing the constructs in Fig 2-1 (which should be a main text figure) would help.

6. Materials & Methods: Animals. The MS would be greatly improved by including a table listing all the strains employed - and including both the formal name (as is already listed) but also the abbreviated name as used spottily in the text, e.g. Etv1-KO.

7. Line 138: “spines” not exactly the right word. Maybe spinal column and associated DRGs?

8. Line 182: It is not exactly clear what was counted in GG cell counts. The text states “Fluorescence around the nucleus...” but how much fluorescence Is not clear. Is just one spot enough to have the cell counted? Were Etv1+ nuclei the same size as Etv1- nuclei? For the counts to be valid, measurements of nuclear size must be included for at least 10 nuclei of each type. If significant size differences exist, counts must be corrected.

9. Line 217: For the Etv1 antibody, what was the antigen and how was specificity validated? Does this antibody stain all forms of Etv1?

10. Line 310: What is Etv1/Er81? Needs a better explanation of what is meant by “Authentic Etv1”.

11. Fig. 2-1 and the accompanying text are very confusing. It is not clear to this reviewer why the different primer sets were used and what the results mean. What is authentic Etv1? This entire section on page 14 needs to be re-written to make this experiment more accessible to the readers.

12. Line 364 and following paragraph: This passage is very confusing. Does not make clear whether animals were tamoxifen-treated. What does the Etv1-cretam + tamoxifen look like at 1-2 days post tamox?

13. Line 383: Axotomy of gustatory nerves causes loss of taste buds. Authors reference John et al 2003 as the earliest reference. However, this was described by many previous authors nearly a century earlier. A more scholarly treatment of this sort of info would be welcome.

14. The sentence at the bottom of p. 17 is also awkwardly written and difficult to follow.

15. Line 399-400: Nearly incomprehensible. Later in the paragraph the authors go off into T1R hetero- and homomers which is not supported by current findings. This might be mentioned as speculation in the discussion, but not here.

16. P. 18. All references to Fig. 8 should be to Fig. 9

17. Line 446: “more or less”?

18. P. 20, lines 454-459. This is also awkwardly written and unclear.

19. P. 22, lines 494-498. Again, what is authentic Etv1? This passage is difficult to understand as written. Then later the paragraph digresses far off course into cancer and other tissues,

20. Figure 7 is very confusing. Is 7A top het and bottom homo, as in B? However, in the legend it says the top is Etv1 Cre crossed with Rosa26 LSL-tom. Are these crosses different then from the cross in 7B? Again the many lines are very confusing but a table may help. Also in Fig. 7 the authors need to explain what KCNQ1 is and what it marks. Similarly the authors need to explain what DDC is (Type III cell marker) and what its use was in the context of this paper. A good place to put this sort of info would be in a table listing all the antibodies employed.
